# Neural network-based model for evaluating inert nodules and volume doubling time in T1 lung adenocarcinoma: a nested case−control study

**DOI:** 10.3389/fonc.2023.1037052

**Published:** 2023-05-24

**Authors:** Bing Wang, Hui Zhang, Wei Li, Siyun Fu, Ye Li, Xiang Gao, Dongpo Wang, Xinjie Yang, Shaofa Xu, Jinghui Wang, Dailun Hou

**Affiliations:** ^1^ Department of Radiology, Beijing Tuberculosis and Thoracic Tumor Research Institute, Beijing Chest Hospital, Capital Medical University, Beijing, China; ^2^ Department of Medical Oncology, Beijing Tuberculosis and Thoracic Tumor Research Institute, Beijing Chest Hospital, Capital Medical University, Beijing, China; ^3^ Cancer Research Center, Beijing Chest Hospital, Capital Medical University, Beijing Tuberculosis and Thoracic Tumor Research Institute, Beijing, China

**Keywords:** neural network, inert nodules, volume doubling time, T1 lung adenocarcinoma, computer tomograph

## Abstract

**Objective:**

The purpose of this study is to establish model for assessing inert nodules predicting nodule volume-doubling.

**Methods:**

A total of 201 patients with T1 lung adenocarcinoma were analysed retrospectively pulmonary nodule information was predicted by an AI pulmonary nodule auxiliary diagnosis system. The nodules were classified into two groups: inert nodules (volume-doubling time (VDT)>600 days n=152) noninert nodules (VDT<600 days n=49). Then taking the clinical imaging features obtained at the first examination as predictive variables the inert nodule judgement model <sn</sn>>(INM) volume-doubling time estimation model (VDTM) were constructed based on a deep learning-based neural network. The performance of the INM was evaluated by the area under the curve (AUC) obtained from receiver operating characteristic (ROC) analysis the performance of the VDTM was evaluated by R^2^(determination coefficient).

**Results:**

The accuracy of the INM in the training and testing cohorts was 81.13% and 77.50%, respectively. The AUC of the INM in the training and testing cohorts was 0.7707 (95% CI 0.6779-0.8636) and 0.7700 (95% CI 0.5988-0.9412), respectively. The INM was effective in identifying inert pulmonary nodules; additionally, the R2 of the VDTM in the training cohort was 0.8008, and that in the testing cohort was 0.6268. The VDTM showed moderate performance in estimating the VDT, which can provide some reference during a patients’ first examination and consultation

**Conclusion:**

The INM and the VDTM based on deep learning can help radiologists and clinicians distinguish among inert nodules and predict the nodule volume-doubling time to accurately treat patients with pulmonary nodules.

## Introduction

With the continuous improvements in health awareness and the wide application of computed tomography (CT) in screening for COVID-19, the detection rate of pulmonary nodules is increasing, greatly affecting the physical and mental health of people. Early lung cancer often appears in the form of pulmonary nodules, which can be divided into solid nodules and subsolid nodules, the latter of which includes pure ground-glass nodules (pGGNs) and mixed GGNs (mGGNs) (1, 2) according to the presence of solid components. The wide application of CT in routine clinical practice enables lung cancer to be detected and treated at a relatively early stage. The National Lung Screening Test (NLST) and Nederland-Leuvens Longkanker Screenings Onderzoek (Nelson) revealed that CT screening is more effective than X-ray screening and can help reduce the mortality of patients. However, most of the positive results found in the screening process are false positives (3, 4), and most of the nodules are benign (5) and need no further treatment. Therefore, in the process of clinical diagnosis and treatment, which nodules need active surgical intervention, which nodules can continue to be followed up and how long they need to be followed up are matters of concern.

In recent years, a new artificial intelligence technology, deep learning systems (DLS), has arisen that can learn image features directly from data. DLS has achieved initial success in detecting pulmonary nodules from chest CT images, and the establishment of a computer-aided diagnosis (CAD) system can help doctors interpret CT images more effectively and accurately (6)Most previous studies have focused on predicting the histological types of nodules or judging whether they are benign or malignant (7–10). The deep learning model shows its potential to be used to accurately identify malignant and invasive subsolid nodules (11).

With the continuous development of deep learning, the clinical application of artificial intelligence pulmonary nodule diagnosis assistant systems is becoming increasingly mature. In many studies, such systems have been shown to provide good effectiveness in assisting the detection of pulmonary nodules (12–14). Some researchers have studied the effect of nodule volume-doubling time on nodules (15–19), while others have explored the growth rate of pulmonary nodules and the natural history of invasive adenocarcinoma (20–22). In a study on the growth of small pulmonary nodules, Xue (23) et al. developed a nomogram based on the combination of radiomics and clinical parameters to predict 2-year growth in the case of uncertain small pulmonary nodules. Diagnostic models based on deep learning can differentiate between benign and malignant pulmonary nodules on chest CT with the same accuracy as daily working radiologists; the uncertain diagnosis is significantly reduced, which can improve confidence in the diagnosis of pulmonary nodules and help clinical decision-making (24).

However, in addition to the nodule size, CT images provide additional information. The British Thoracic Society’s guidelines on pulmonary nodules recommend measuring volume rather than diameter because it is less prone to intra-observer and inter-observer variation. In the context of pulmonary nodules, volume doubling time is a key indicator of malignant tumors.

In our study, we developed an inert nodule judgement model (INM) and a volume-doubling time estimation model (VDTM) based on a convolutional neural networks (CNN) algorithm to evaluate the inert-growth trend of pulmonary nodules, analyse the properties of pulmonary nodules, and manage pulmonary nodules scientifically.

## Materials and methods

### Patients

**Figure 1:**
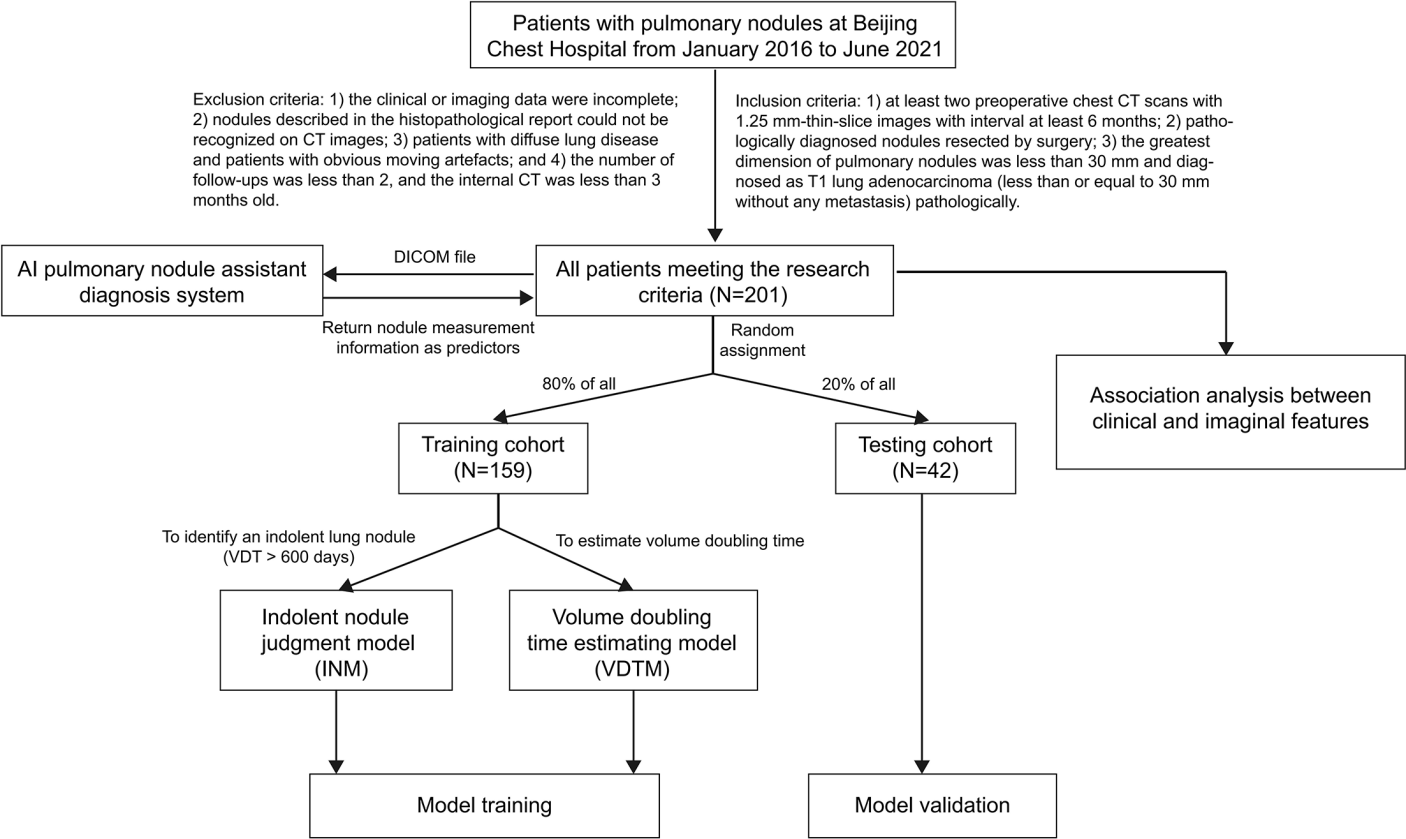
Flow chart of this study. VDT, volume doubling time.

This study was designed as a nested case−control study. A total of 201 patients with pulmonary nodules who underwent surgery at Beijing Chest Hospital affiliated with Capital Medical University from January 2016 to June 2021 were selected. The inclusion criteria were as follows: 1) at least two preoperative chest CT scans with 1.25-mm slice thickness, and with an interval of at least 6 months; 2) nodules confirmed by histology and surgery as T1 stage lung adenocarcinoma without blood or lymph node metastases; 3) the greatest diameter of pulmonary nodules was less than 30 mm. The exclusion criteria were as follows: 1) the clinical or imaging data were incomplete; 2) the GGNs described in the histopathological report could not be recognized on CT images; 3) patients with diffuse lung disease and patients with obvious moving artefacts; and 4) the number of follow-ups was less than 2, and the internal of two preoperative chest CT was less than 6 months. We collected all the preoperative CT images, clinical features and postoperative pathological data of the patients, and the results are shown in **Figure 1**.

### CT scan

All patients were scanned using a 16-cm wide detector CT (Revolution 256, GE Healthcare, Milwaukee, USA. First, a CT plain scan was carried out. The scanning range was from the top of the lung to the bottom of the lung, and then the patient was injected intravenously with iohexol concentration of 300mgI/mL, and the dose was calculated according to the patient’s body weight multiplied by 1.2-2 mL/kg. The injection flow rate was 3.0 mL/s. After injection, the enhanced scan began with a delay of 20 s and 50 s, and the relevant parameters were set as follows: tube voltage 120 kV, tube current set for automatic adjustment, slice spacing 1.25 mm, and pitch 1.0. Patients fasted for 5 hours before the enhanced CT scan. The standard reconstruction method was used to reconstruct the image.

### Nodule feature analysis

The CT images of all patients (slice thickness and interval are 1.25 mm) were imported into the InferRead CT Lung (Infervision Medical Technology Co., Ltd.) in DICOM format, which was used to obtain the nodule measurement information. The data included CT value (HU), longest diameter, shortest diameter, mean diameter, nodular volume, nodular density analysis, proportion of solid components, malignant risk assessment, nodular morphology, edge, internal structure, nodular volume doubling time (VDT), and so on.

### Pathological diagnosis

The pathological subtypes of pulmonary nodules in all patients were classified according to the WHO Thoracic Tumours Classification. The diagnosis of all pathological specimens of each case was confirmed by at least two experienced pathologists, and whenever there was a disagreement, a consensus was reached through mutual discussion or consultation with a third pathologist.

### Inert nodule judgement model

The British Thoracic Society guidelines on the investigation and management of pulmonary nodules note that a VDT > 600 days means a very low risk of malignancy, and the patient can even be discharged (25). The results of previous studies supported a similar opinion (26, 27). Although some traditional views suggest that a nodule with a VDT > 400 days tends to be indolent, we conservatively chose 600 days as the cut-off number of days for defining an inert lung nodule (26–29).

All patients were randomly allocated to the training (159) or testing cohort (42). Clinical characteristics and imaging features extracted during the first examination based on the AI pulmonary nodule assistant diagnosis system were selected as predictive variables, including age, sex, smoking status, nodule location, nodule type, minimum CT value, maximum CT value, average CT value, kurtosis (the kurtosis of nodule’s CT values), skewness (the skewness of nodule’s CT values), CT longest diameter, CT shortest diameter, CT average diameter, volume of nodule, proportion of solid components, nodule shape, lobulation, spiculation and pleural retraction.

Numerical variables were normalized (subtracted the standard deviation and divided by the mean), while categorical variables were transformed to one-hot encoding (which means using numbers instead of characters to reflect the variables, such as replacing “Female” with “1”).

The training cohort was then utilized to create an inert nodule judgement model (INM) based on a neural network estimate of whether a nodule would be indolent (VDT > 600 days). Dropout slices and the testing cohort were applied to avoid overfitting. Given the presence of unbalanced data (only 49 people’s VDT ≤ 600 days, but 152 patients’ VDT>600 days), positive outcomes were provided greater weighting to more accurately train the model.

The area under the receiver operating characteristic (ROC) curve (AUC) was used to evaluate the classification performance (inert nodule or noninert nodule) of the INM.

The neural network was conducted with python 3.9.7 and its packages pytorch, torchtuples, numpy, pandas and sklearn, while the package matplotlib was used to help visualize the training process (30–35). R and its packages pROC and ggplot2 were used to plot the ROC curves (36–38).

### Volume-doubling time estimation model

Using the clinical and CT features in the INM, we built another neural network to estimate the VDTM. As discussed above, the training cohort data were used to train the VDTM, and the testing cohort was used to externally validate the model. The R^2^, also known as the coefficient of determination of the model, was selected as the main performance evaluation indicator since a regression task was involved. Better model predictive performance was indicated by an R^2^ value closer to 1. We achieved the above work mainly with Python and sklearn.

### Association between clinical and CT features

Association analysis of the clinical and CT features was conducted to explore the potentially intrinsic relationships between the two groups of features. In this process, the Spearman correlation coefficient was used with a heatmap and chord diagram to visualize the results. We used the R packages corrplot and circlize to generate the heatmap and the chord diagram (39, 40).

### Statistical analysis

Numerical data with normal and nonnormal distributions were compared using Student’s t test and the Wilcoxon test, respectively. A two-sided P < 0.05 was considered to be statistically significant. All statistical analyses were completed with R 4.1.2 and the package epiDisplay (41).

## Results

### Patient characteristics

A total of 201 lung nodules of T1 lung adenocarcinoma patients were included. Forty-nine patients had a VDT equal to or less than 600 days, while 152 patients had a VDT greater than 600 days (the VDT was predicted by artificial intelligence assistant software). The two groups (VDT > 600 days and ≤ 600 days) of patients demonstrated differences in histology type, pathological subtypes of pulmonary nodules, nodule type(classified by density), kurtosis of nodule CT values, pathological size, CT longest diameter, CT shortest diameter, CT average diameter, nodule shape, lobulation, spiculation and pleural retraction (**Table 1**).

**Table 1:** The comparison of clinical and CT features between patients with nodule VDT≤600d and>600d.

	VDT≤600d	VDT≤600d	Statistical method	P value	
	(N=49)	(N=152)			
Age			Wilcoxon	0.3903	
Median (IQR)	60 (50, 63)	56 (48.75, 64)		
Sex			Chi-square	0.0731	
Female	31 (63.27)	116 (76.32)		
Male	18 (36.73)	36 (23.68)		
Smoke			Chi-square	0.1481	
No	40 (81.63)	136 (89.47)		
Yes	9 (18.37)	16 (10.53)		
Pathology			Fisher's exact	0.0427*	
AAH	0 (0)	2 (1.32)		
AIS	3 (6.12)	16 (10.53)		
MIA	15 (30.61)	73 (48.03)		
IAC	31 (63.27)	61 (40.13)		
Component			Fisher's exact	0.0064**	
Acinar predominant	15 (30.61)	67 (44.08)		
Lepidic predominant	7 (14.29)	19 (12.50)		
Micropapillary predominant	2 (4.08)	0 (0)		
Papillary predominant	13 (26.53)	25 (16.45)		
Solid predominant	3 (6.12)	1 (0.66)		
Unknown	9 (18.37)	40 (26.32)		
Location			Chi-square	0.6754	
Superior lobe of left lung	10 (20.41)	38 (25.00)		
Inferior lobe of left lung	8 (16.33)	15 (9.87)		
Superior lobe of right lung	20 (40.82)	62 (40.79)		
Middle lobe of right lung	4 (8.16)	9 (5.92)		
Inferior lobe of right lung	7 (14.29)	28 (18.42)		
Surgical method			Fisher's exact	0.424	
Wedge resection	17 (34.69)	54 (35.53)		
Segmentectomy	5 (10.20)	21 (13.82)		
Lobectomy	26 (53.06)	77 (50.66)		
Pneumonectomy	1 (2.04)	0 (0)		
Mutation			Fisher's exact	0.4544	
EGFR	15 (30.61)	37 (24.34)		
KRAS	0 (0)	7 (4.61)		
ROS1	0 (0)	1 (0.66)		
ALK	1 (2.04)	1 (0.66)		
HER2	0 (0)	4 (2.63)		
MET14	0 (0)	1 (0.66)		
BRAF	1 (2.04)	1 (0.66)		
Negative	5 (10.20)	18 (11.84)		
Unknown	27 (55.10)	82 (53.95)		
Nodule type			Chi-square	0.0447*	
Mixed GGN	18 (36.73)	72 (47.37)		
Pure GGN	20 (40.82)	66 (43.42)		
Solid nodule	11 (22.45)	14 (9.21)		
Minimum CT value (HU)			Wilcoxon	0.137	
Median (IQR)	-672 (-743, -511)	-718 (-757.25, -619.5)		
Maximum CT value (HU)			Wilcoxon	0.3609	
Median (IQR)	54 (-153, 170)	-15.5 (-194.75, 137)		
Average CT value (HU)			Wilcoxon	0.1702	
Median (IQR)	-449 (-637, -146)	-535.5 (-612, -352.25)		
Kurtosis			Wilcoxon	0.0418*	
Median (IQR)	0.36 (0.08, 0.66)	0.52 (0.20, 0.88)		
Skewness			Wilcoxon	0.2402	
Median (IQR)	-0.76 (-1.01, 0.04)	-0.5 (-0.97, 0.54)		
Pathological size (mm)			Wilcoxon	0.0022**	
Median (IQR)	11 (7, 15)	8 (5.75, 11)		
CT longest diameter (mm)			Chi-square	0.0053**	
≤ 8	11 (22.45)	55 (36.18)		
≤ 10	5 (10.2)	32 (21.05)		
≤ 20	23 (46.94)	55 (36.18)		
≤ 30	10 (20.41)	10 (6.58)		
CT shortest diameter (mm)			Wilcoxon	0.0176*	
Median (IQR)	11 (6, 13)	8 (6, 10)		
CT average diameter (mm)			Wilcoxon	0.0085**	
Median (IQR)	12 (7, 16)	9 (7, 13)		
Volume of nodule (mm^3^)			Wilcoxon	0.7972	
Median (IQR)	213.55 (93.67, 695.49)	248.2 (129.66, 521.85)		
Proportion of solid ingredients			Wilcoxon	0.0739	
Median (IQR)	1.86 (0, 53.44)	0.61 (0, 9.43)		
Shape of nodule			Chi-square	< 0.001***	
Irregular	14 (28.57)	13 (8.55)		
Regular	35 (71.43)	139 (91.45)		
Lobulation			Chi-square	0.0298*	
No	22 (44.90)	95 (62.50)		
Yes	27 (55.10)	57 (37.50)		
Spiculation			Chi-square	0.0041**	
No	22 (44.90)	103 (67.76)		
Yes	27 (55.10)	49 (32.24)		
Pleural retraction			Chi-square	0.0133*	
No	36 (73.47)	134 (88.16)		
Yes	13 (26.53)	18 (11.84)		

VDT, volume doubling time; IQR, interquartile range; AAH, atypical adenomatous hyperplasia; AIS, adenocarcinoma *in situ*; MIA, minimally invasive adenocarcinoma; IAC, invasive adenocarcinoma; GGN, ground glass nodule; CT, computed tomography; HU, Hounsfield unit; Kurtosis, the kurtosis of nodule’s CT values; Skewness, the skewness of nodule’s CT values. Missing values were not analyzed statistically. *P<0.05, **P<0.01, ***P<0.001.

### Identification of indolent lung nodules

The INM was designed to identify indolent lung nodules, including 3 hidden slices (16, 4 and 2 nodes) and dropout slices. Clinical and CT features acquired at the first CT scan were extracted as the predictive variables, including age, sex, smoking status, nodule location, nodule type, minimum CT value, maximum CT value, average CT value, kurtosis (of the nodule’s CT values), skewness (of the nodule’s CT values), CT longest diameter, CT shortest diameter, CT average diameter, nodule volume, proportion of solid components, nodule shape, lobulation, spiculation and pleural retraction.

**Figure 2:**
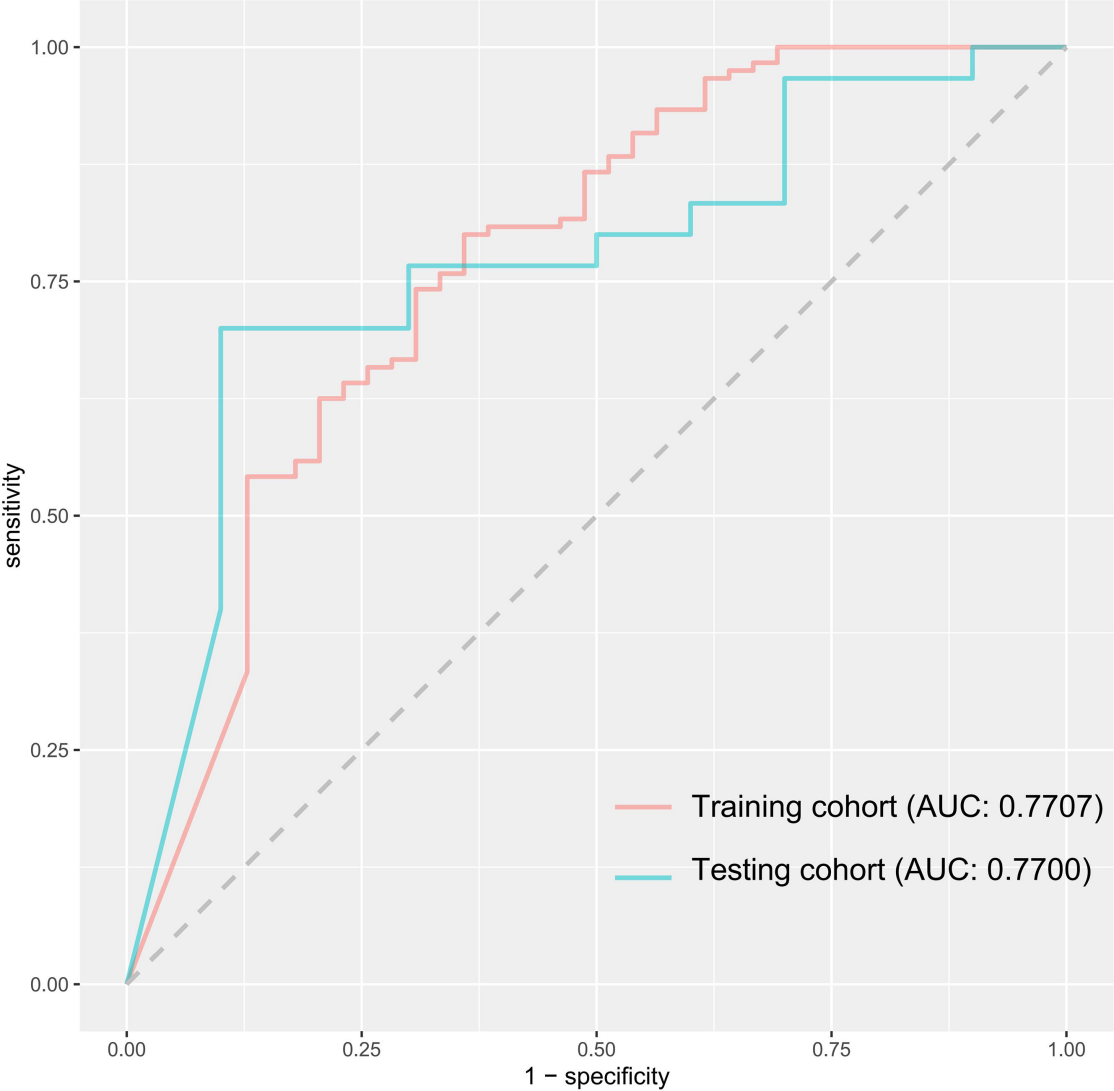
The receiver operating characteristic curve of indolent nodule judgment model (INM). AUC, area under receiver operating characteristic curve.

The INM had an accuracy of 81.13% and 77.50% in training and testing cohorts, respectively. The AUCs were 0.7707 (95% confidence interval, CI, 0.6779-0.8636 by the DeLong method) in the training cohort and 0.7700 (95% CI, 0.5988-0.9412 by DeLong method) in the testing cohort (**Figure 2**). The INM showed a slightly satisfying performance in identifying indolent lung nodules. With patients’ clinical and CT features as the input, the INM returned a speculative probability that a nodule would be indolent. It would help doctors and patients decide whether a nodule belonged to the indolent category simply on the basis of the first-time CT scan. The codes of the INM have been uploaded in **Additional File 1**, and its detailed parameters have been saved in **Additional File 2**.

### Estimation of volume doubling time

To obtain an approximate VDT simply on the basis of the patient’s first CT scan, we built the VDTM. The same clinical and CT features used in the INM were chosen as predictive variables. The R^2^ of the VDTM was 0.8008 in the training cohort and 0.6268 in the testing cohort. Neural networks were used for classification tasks (such as “malignant” or “benign”), and their performance on regression tasks (for estimating a numeric result, such as how many days) was moderate. The VDTM showed a moderate performance in estimating VDT, but it could still offer some references at a patient’s first examination and consultation. The codes of the VDTM have been uploaded in **Additional File 3**, and its detailed parameters have been saved in **Additional File 4**.

### Association between clinical and CT features

**Figure 3:**
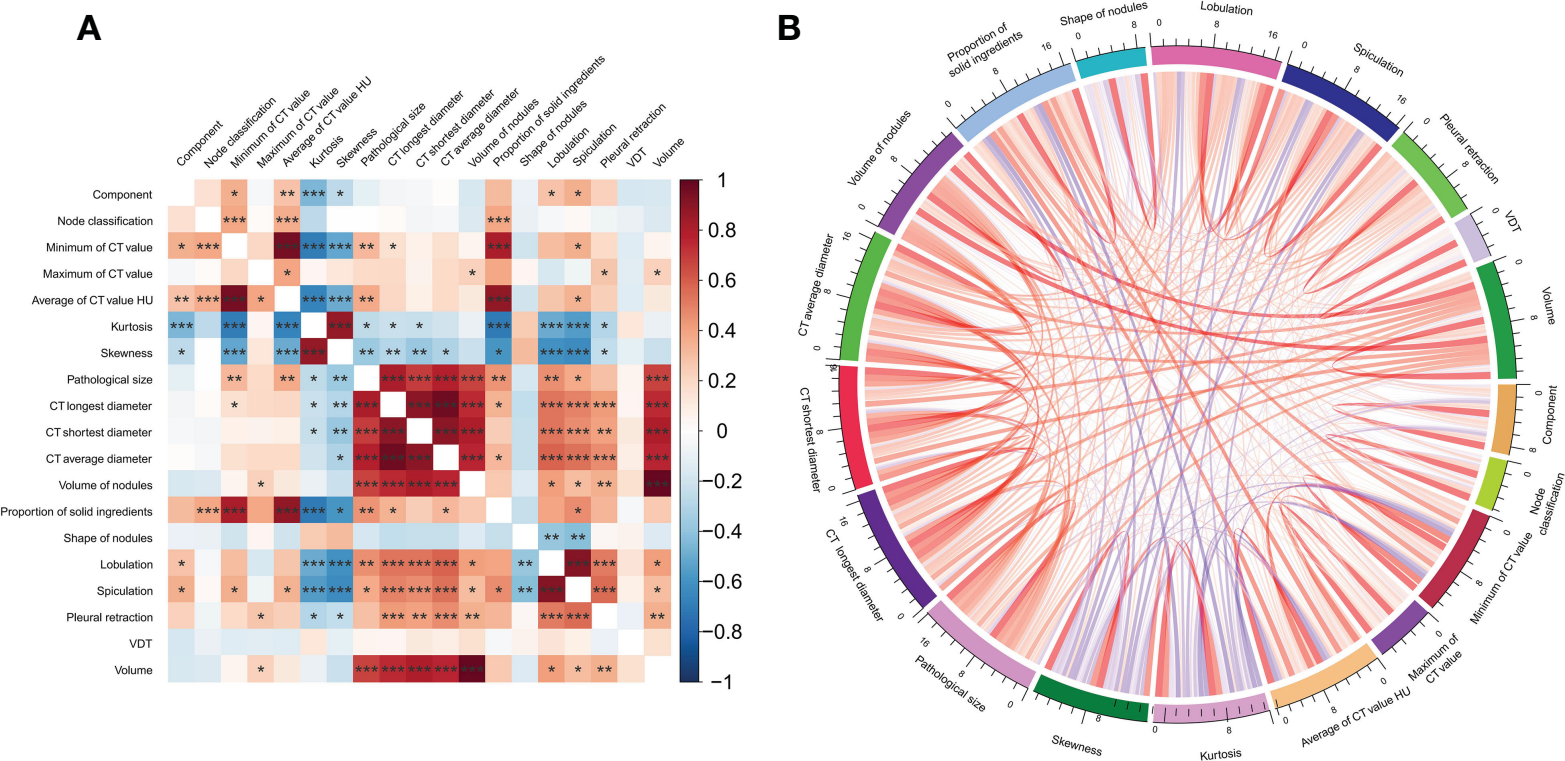
The association analysis between clinical and imaginal features visualized by **(A)** heatmap plotting. **(B)** Chord diagram.

Our primary aim was to determine whether the CT features extracted during the first CT scan could reveal later pathological features. The association analysis between pathological features and certain clinical features showed that the pathology subtype was associated with the minimum CT value, average CT value, kurtosis, skewness, lobulation,spiculation,pleural retraction. Invasive histology subtypes was related to the maximum CT value, kurtosis, skewness, CT longest diameter, CT shortest diameter, CT average diameter, nodule volume, lobulation, and spiculation **(** **Figure 3** **).**

## Discussion

In this study, we developed a deep learning prediction model to distinguish between inert nodules and noninert nodules based on the initial CT characteristics. The deep learning model was the first used for predicting the inert-growth trend of pulmonary nodules. The deep learning prediction model yielded good prediction performance, with AUCs of 0.7707 and 0.7700 in the training and testing cohorts, respectively.

In our study, the analysis of the clinical characteristics of patients with T1 lung adenocarcinoma showed that there was no clear relationship between nodule inert-growth status and patient age and sex, and there was no significant difference between smoking and lung nodule inert-growth status. This is not consistent with previous studies showing that smoking is an independent risk factor for lung cancer (42). Research on artificial intelligence-aided image diagnosis systems has been reported in the literature. In this study, the extraction of nodule information was based on an artificial intelligence-aided diagnosis system. The continuous improvement of the system in clinical application can provide great help for reducing the number of missed diagnoses. The inert growth of pulmonary nodules was related to the pathology subtype, nodular type, CT kurtosis, CT longest diameter, CT shortest diameter, CT mean diameter, nodule shape, lobulation, spiculation, pleural retraction and so on. Among nodules of different diameters, the VDT was significantly shortened with increasing nodular diameter. In patients with nodular diameters less than 20mm, nodular VDT showed a slow growth process. This coincides with the view that persistent pGGNs exhibit an inert growth process, and pGGNs with lobulation and a larger initial diameter, volume and mass grow more easily. However, our results showed that more characteristics were related to the inert growth of nodules, probably because the nodules analysed in our study were not only pGGNs but also included mGGNs and solid nodules. In this study, We did not classify according to the pathological subtypes of pulmonary nodules future studies should provide supplementary data.

After the imaging parameters were read out by the AI pulmonary nodule assistant diagnosis system, we integrated them with the patient’s clinical data for clinical modelling. Compared with the traditional model constructed by logistic regression, the machine learning algorithm has more advantages in solving classification problems. As the advanced algorithm in the field of machine learning, neural networks have attracted wide attention in the field of medicine in recent years. Deep learning neural networks, which are inspired by the biological nervous system, usually contain multiple hidden slices and multiple nodes between slices. It predicts the final event, calculates the loss between the predicted value and the actual value, reverses the weight of each slice and node, and constantly learns the characteristics of the relationship between the prediction variables and outcome variables. The weights between slices are constantly updated, and due to the addition of activation functions, the neural network algorithm is especially suitable for solving nonlinear problems. Traditional models (logistic, Cox regression, etc.) are based on linear hypotheses, so real-world data, especially clinical and imaging data, are more suitable for neural network calculations. Finally, we also chose neural network modelling to determine whether the nodules were inert and to roughly estimate the VDT.

### Inert nodule judgement model

In our study, we constructed an INM based on initial CT; it had an accuracy of 81.13% in the training cohort and 77.50% in the testing cohort. Combining clinical variables and the INM resulted in a good ability to distinguish inert nodules from noninert nodules. In previous studies, the focus was mainly on distinguishing between benign and malignant nodules and between noninvasive and invasive nodules. Xu et al. (43) found that a DL model trained on VOIs from continuous CT images had good predictive performance in distinguishing noninvasive GGNs from invasive GGNs. KimH et al. (7) developed a deep learning model using 2.5D and 3D CT images of 525 preoperative patients to distinguish IACs among subsolid nodules (SSN) for surgical candidates. Chang et al. (16) found that approximately 90% of the pGGNs detected by screening did not grow during long-term follow-up in patients with no history of malignant tumours, and most of the growing nodules had an inert clinical course. For pGGN, a strategy of long-term follow-up and selective surgery for growing nodules should be considered. In contrast to previous studies, we did not classify nodules according to previous clinicopathological classification, degree of malignancy or solid components of nodules. We proposed to take the VDT as the research target, regardless of histology or density type to which the nodules belong and of whether they increase or decrease in size during follow-up. Our research is intended to assist doctors and patients in judging the growth trend of nodules from the first CT scan. Patients should be given reasonable follow-up advice for the pulmonary nodules.

### Volume-doubling time estimation model

In previous studies, volume doubling time has often been used as an index to evaluate the properties of nodules. Tumour VDT is one of the indicators used to evaluate tumour growth and is closely related to the concept of overdiagnosis in lung cancer screening. Nelson studies have shown that the VDT can be used to distinguish invasive tumours from inert tumours (29). Heuvelmans et al. (13) found that in the baseline screening of the Nelson test, all malignant, fast-growing pulmonary nodules referred after 3-month follow-up CT had a VDT ≤ 232 days. Lowering the cut off VDT may reduce false-positive referrals. Song et al. found that (11) nodules less than or equal to 5 mm showed longer VDT and mass doubling time(MDT) than larger than 5 mm. In our study, we constructed a VDTM based on the clinical and imaging features used in the INM as predictive variables. The neural network we used for classification tasks (such as “malignant” or “benign”) was excellent, but its performance in regression tasks (to estimate a numerical result, such as how many days) was not good. The VDTM showed moderate performance in estimating VDT, but it could still provide some reference in patients’ first examination and consultation.

Our results showed that the evaluation model of inert growth of pulmonary nodules based on deep learning could improve the accuracy of diagnosis and helped the treatment decision-making. In this study, our proposed model was trained and tested with histopathologically confirmed T1 lung adenocarcinoma nodules, and clinical features and multiple follow-up CT features were added to the deep learning model. Compared with the method of establishing a single deep learning model or using clinical characteristics, the evaluation ability of our method was further enhanced.

There are some limitations in this study. First, this study was retrospective in nature, and the sample size was relatively small. Therefore, selection bias was unavoidable. Second, this study did not include blood markers during follow-up of the nodules, and future studies should involve a larger sample size and multicentre samples to increase the generalizability of the models. Third, in our study, only the inert-growth prediction model of lung nodules in T1 lung adenocarcinoma, models applied to other stages and other types of lung cancer to be further developed.

In summary, the nodule growth prediction model based on the VDT of pulmonary nodules had important value in assessing the properties of nodules. Our study allowed us to contribute to identifying potential early lung cancer by integrating current clinical and multiple follow-up CT results with deep learning neural networks.

## Data availability statement

The original contributions presented in the study are included in the article/**Supplementary Material**. Further inquiries can be directed to the corresponding authors.

## Ethics statement

The studies involving human participants were reviewed and approved by Beijing Chest Hospital, Capital Medical University (JYS-2021-026). The patients/participants provided their written informed consent to participate in this study.

## Author contributions

BW, HZ, WL, SX,JW and DH contributed to conception and design of the study. BW, YL,DH and DW organized the database. HZ, WL, SF, XG and XY performed the statistical analysis. BW, HZ and WL wrote the first draft of the manuscript. SX, JW and DH wrote sections of the manuscript. All authors contributed to manuscript revision, read, and approved the submitted version.
