# Supplementary material for: Neural network-based model for evaluating inert nodules and volume doubling time in T1 lung adenocarcinoma: a nested case−control study
Source: Front Oncol. 2023 May 24;13:1037052. doi: 10.3389/fonc.2023.1037052 (PMC10244560; doi:10.3389/fonc.2023.1037052)
Supplement: Supplementary file 1 [file DataSheet_1.pdf]

In [1]:

```
import torch
import torchtuples as tt

import torchtuples as pd
import numpy as np

from sklearn.preprocessing import StandardScaler, LabelEncoder
from sklearn.model_selection import train_test_split
```

In [2]:

```
from sklearn.metrics import roc_auc_score
```

In [3]:

```
df_raw = pd.read_csv('model_data.csv', na_values="Unknown")
df_raw = pd.DataFrame(df_raw)
df_raw = df_raw.dropna()
df_raw.head()
```

Out[3]:

	Age	Sex	Smoke	Location	Node classification	Minimum of CT value	Maximum of CT value	Average of CT value(HU)	Kurtosis	S
**0**	50	Female	No	Superior lobe of right lung	Mixed ground-glass nodule	-765	22	-563.0	0.19	
**1**	43	Female	No	Superior lobe of right lung	Solid nodule	-361	54	-146.0	-0.05	
**2**	70	Female	No	Superior lobe of right lung	Mixed ground-glass nodule	-528	25	-341.0	0.31	
**3**	37	Female	No	Superior lobe of right lung	Mixed ground-glass nodule	-591	-8	-382.0	0.21	
**4**	71	Female	No	Superior lobe of right lung	Mixed ground-glass nodule	-538	78	-258.0	0.01	

In [4]:

```
# take out y
df_raw_y = df_raw["VDT"]
df_raw_y.head()
```

Out[4]:

```
0    1
1    0
2    1
3    1
4    1
Name: VDT, dtype: int64
```

In [5]:

```
df_raw.columns
```

Out[5]:

```
Index(['Age', 'Sex', 'Smoke', 'Location', 'Node classification',
       'Minimum of CT value', 'Maximum of CT value', 'Average of CT value(HU)',
       'Kurtosis', 'Skewness', 'CT longest diameter', 'CT shortes diameter',
       'CT average diameter', 'Volume of nodules',
       'Proportion of solid ingredients', 'Shape of nodules', 'Lobulation',
       'Spiculation', 'Pleural retraction', 'VDT'],
      dtype='object')
```

In [6]:

```
# take out x
df_raw_x = df_raw[['Age', 'Sex', 'Smoke', 'Location', 'Node classification',
       'Minimum of CT value', 'Maximum of CT value', 'Average of CT value(HU)',
       'Kurtosis', 'Skewness', 'CT longest diameter', 'CT shortes diameter',
       'CT average diameter', 'Volume of nodules',
       'Proportion of solid ingredients', 'Shape of nodules', 'Lobulation',
       'Spiculation', 'Pleural retraction']]
df_raw_x.head()
```

Out[6]:

	Age	Sex	Smoke	Location	Node classification	Minimum of CT value	Maximum of CT value	Average of CT value(HU)	Kurtosis	S
**0**	50	Female	No	Superior lobe of right lung	Mixed ground-glass nodule	-765	22	-563.0	0.19	
**1**	43	Female	No	Superior lobe of right lung	Solid nodule	-361	54	-146.0	-0.05	
**2**	70	Female	No	Superior lobe of right lung	Mixed ground-glass nodule	-528	25	-341.0	0.31	
**3**	37	Female	No	Superior lobe of right lung	Mixed ground-glass nodule	-591	-8	-382.0	0.21	
**4**	71	Female	No	Superior lobe of right lung	Mixed ground-glass nodule	-538	78	-258.0	0.01	

In [7]:

```
cat_var = ['Sex', 'Smoke', 'Location', 'Node classification',
       'Shape of nodules', 'Lobulation',
       'Spiculation', 'Pleural retraction']
num_var = ['Age', 'Minimum of CT value', 'Maximum of CT value', 'Average of CT value(HU)',
       'Kurtosis', 'Skewness', 'CT longest diameter', 'CT shortes diameter',
       'CT average diameter', 'Volume of nodules',
       'Proportion of solid ingredients',]
```

In [8]:

```
# to label encoding categorical variables
df_raw_x = pd.DataFrame(df_raw_x)
df_raw_x = pd.get_dummies(df_raw_x, columns=cat_var)

df_raw_x.head()
```

Out[8]:

	Age	Minimum of CT value	Maximum of CT value	Average of CT value(HU)	Kurtosis	Skewness	CT longest diameter	CT shortes diameter	CT average diameter
**0**	50	-765	22	-563.0	0.19	-1.19	14	12	13
**1**	43	-361	54	-146.0	-0.05	-1.11	13	11	12
**2**	70	-528	25	-341.0	0.31	-0.86	11	7	9
**3**	37	-591	-8	-382.0	0.21	-0.93	14	11	13
**4**	71	-538	78	-258.0	0.01	-1.19	30	25	28

5 rows × 31 columns

In [9]:

```
x_train_raw, x_test_raw, y_train, y_test = train_test_split(df_raw_x, df_raw_y, test_size=0.2, ra
```

In [10]:

```
x_train_raw[num_var].describe()
```

Out[10]:

	Age	Minimum of CT value	Maximum of CT value	Average of CT value(HU)	Kurtosis	Skewness	CT longes diamete
**count**	159.000000	159.000000	159.000000	159.000000	159.000000	159.000000	159.00000
**mean**	54.993711	-648.584906	10.232704	-467.811321	0.558176	0.012516	12.29559
**std**	10.849078	160.730386	319.627437	204.802813	0.536682	2.067507	5.77155
**min**	30.000000	-845.000000	-649.000000	-809.000000	-1.130000	-1.250000	5.00000
**25%**	47.500000	-754.000000	-185.000000	-621.000000	0.150000	-1.000000	8.00000
**50%**	57.000000	-709.000000	-8.000000	-530.000000	0.470000	-0.500000	10.00000
**75%**	63.000000	-569.000000	138.000000	-333.000000	0.860000	0.500000	15.50000
**max**	76.000000	-92.000000	1886.000000	77.000000	3.250000	21.850000	30.00000

In [11]:

```
x_train = x_train_raw.copy()
x_train["Age"] = (x_train_raw["Age"] - 54.993711) / 10.849078
x_train['Minimum of CT value'] = (x_train_raw['Minimum of CT value'] + 648.584906) / 160.730386
x_train['Maximum of CT value'] = (x_train_raw['Maximum of CT value'] - 10.232704) / 319.627437
x_train['Average of CT value(HU)'] = (x_train_raw['Average of CT value(HU)'] + 467.811321) / 204.
x_train['Kurtosis'] = (x_train_raw['Kurtosis'] - 0.558176) / 0.536682
x_train['Skewness'] = (x_train_raw['Skewness'] - 0.012516) / 2.067507
x_train['CT longest diameter'] = (x_train_raw['CT longest diameter'] - 12.295597) / 5.771551
x_train['CT shortes diameter'] = (x_train_raw['CT shortes diameter'] - 9.440252) / 4.265836
x_train['CT average diameter'] = (x_train_raw['CT average diameter'] - 11.157233) / 5.473220
x_train['Volume of nodules'] = (x_train_raw['Volume of nodules'] - 449.534906) / 603.666116
x_train["Proportion of solid ingredients"] = (x_train_raw["Proportion of solid ingredients"] - 13


x_test = x_test_raw.copy()
x_test["Age"] = (x_test_raw["Age"] - 54.993711) / 10.849078
x_test['Minimum of CT value'] = (x_test_raw['Minimum of CT value'] + 648.584906) / 160.730386
x_test['Maximum of CT value'] = (x_test_raw['Maximum of CT value'] - 10.232704) / 319.627437
x_test['Average of CT value(HU)'] = (x_test_raw['Average of CT value(HU)'] + 467.811321) / 204.8[illegible]
x_test['Kurtosis'] = (x_test_raw['Kurtosis'] - 0.558176) / 0.536682
x_test['Skewness'] = (x_test_raw['Skewness'] - 0.012516) / 2.067507
x_test['CT longest diameter'] = (x_test_raw['CT longest diameter'] - 12.295597) / 5.771551
x_test['CT shortes diameter'] = (x_test_raw['CT shortes diameter'] - 9.440252) / 4.265836
x_test['CT average diameter'] = (x_test_raw['CT average diameter'] - 11.157233) / 5.473220
x_test['Volume of nodules'] = (x_test_raw['Volume of nodules'] - 449.534906) / 603.666116
x_test["Proportion of solid ingredients"] = (x_test_raw["Proportion of solid ingredients"] - 13.7
```

In [12]:

```
x_train_use = np.array(x_train, dtype=np.float32)
x_test_use = np.array(x_test, dtype=np.float32)
y_train_use = np.array(y_train, dtype=np.float32).reshape(-1,1)
y_test_use = np.array(y_test, dtype=np.float32).reshape(-1,1)
```

In [13]:

```
import torchtuples as tt
import matplotlib.pyplot as plt
from torchtuples.practical import MLPVanilla, accuracy_binary
```

In [20]:

```
in_features = x_train.shape[1]
num_nodes = [16,4,2]
out_features = 1
batch_norm = True
dropout = 0.4
output_bias = False
epochs = 100
callbacks = [tt.callbacks.EarlyStopping(patience=30)]
metrics = {'acc': accuracy_binary}

net = MLPVanilla(in_features, num_nodes, out_features, batch_norm, dropout, output_bias=output_bi
loss = torch.nn.BCEWithLogitsLoss(pos_weight=torch.tensor([0.6]))
optimizer = tt.optim.Adam(lr=0.01)
model = tt.Model(net, loss, optimizer)

log = model.fit(x_train_use, y_train_use, batch_size=32, epochs=epochs, callbacks = callbacks, me
                val_data=(x_test_use, y_test_use))
```

0: [0s / 0s], train_loss: 0.5719, train_acc: 0.5855, v
al_loss: 0.4477, val_acc: 0.8000
1: [0s / 0s], train_loss: 0.5248, train_acc: 0.6732, v
al_loss: 0.4420, val_acc: 0.7750
2: [0s / 0s], train_loss: 0.5117, train_acc: 0.6292, v
al_loss: 0.4408, val_acc: 0.7750
3: [0s / 0s], train_loss: 0.4698, train_acc: 0.7048, v
al_loss: 0.4370, val_acc: 0.8000
4: [0s / 0s], train_loss: 0.4438, train_acc: 0.7294, v
al_loss: 0.4368, val_acc: 0.8000
5: [0s / 0s], train_loss: 0.4718, train_acc: 0.7349, v
al_loss: 0.4371, val_acc: 0.8000
6: [0s / 0s], train_loss: 0.4297, train_acc: 0.7554, v
al_loss: 0.4344, val_acc: 0.7750
7: [0s / 0s], train_loss: 0.4195, train_acc: 0.7923, v
al_loss: 0.4348, val_acc: 0.7750
8: [0s / 0s], train_loss: 0.4562, train_acc: 0.7359, v
al_loss: 0.4366, val_acc: 0.7750
9: [0s / 0s], train_loss: 0.4275, train_acc: 0.7236, v
al_loss: 0.4357, val_acc: 0.7750
10: [0s / 0s], train_loss: 0.4338, train_acc: 0.7550, v
al_loss: 0.4350, val_acc: 0.7750
11: [0s / 0s], train_loss: 0.4447, train_acc: 0.7875, v
al_loss: 0.4388, val_acc: 0.7500
12: [0s / 0s], train_loss: 0.4153, train_acc: 0.7990, v
al_loss: 0.4385, val_acc: 0.7500
13: [0s / 0s], train_loss: 0.4253, train_acc: 0.7613, v
al_loss: 0.4383, val_acc: 0.7500
14: [0s / 0s], train_loss: 0.4435, train_acc: 0.7669, v
al_loss: 0.4409, val_acc: 0.7500
15: [0s / 0s], train_loss: 0.4059, train_acc: 0.7915, v
al_loss: 0.4457, val_acc: 0.7500
16: [0s / 0s], train_loss: 0.4320, train_acc: 0.7617, v
al_loss: 0.4469, val_acc: 0.7500
17: [0s / 0s], train_loss: 0.3995, train_acc: 0.8107, v
al_loss: 0.4453, val_acc: 0.7500
18: [0s / 0s], train_loss: 0.4264, train_acc: 0.7859, v
al_loss: 0.4453, val_acc: 0.7250
19: [0s / 0s], train_loss: 0.4352, train_acc: 0.7746, v
al_loss: 0.4457, val_acc: 0.7250
20: [0s / 0s], train_loss: 0.4225, train_acc: 0.7921, v
al_loss: 0.4454, val_acc: 0.7250
21: [0s / 0s], train_loss: 0.4308, train_acc: 0.7546, v
al_loss: 0.4418, val_acc: 0.7000
22: [0s / 0s], train_loss: 0.4021, train_acc: 0.7613, v
al_loss: 0.4413, val_acc: 0.7000
23: [0s / 0s], train_loss: 0.4366, train_acc: 0.7677, v
al_loss: 0.4431, val_acc: 0.7250
24: [0s / 0s], train_loss: 0.4141, train_acc: 0.7861, v
al_loss: 0.4452, val_acc: 0.7250
25: [0s / 0s], train_loss: 0.3973, train_acc: 0.7863, v
al_loss: 0.4431, val_acc: 0.7750
26: [0s / 0s], train_loss: 0.4222, train_acc: 0.7732, v
al_loss: 0.4400, val_acc: 0.7750
27: [0s / 0s], train_loss: 0.4020, train_acc: 0.7990, v
al_loss: 0.4436, val_acc: 0.7500
28: [0s / 0s], train_loss: 0.4084, train_acc: 0.7679, v
al_loss: 0.4444, val_acc: 0.7500
29: [0s / 0s], train_loss: 0.3988, train_acc: 0.7861, v
al_loss: 0.4494, val_acc: 0.7500
30: [0s / 0s], train_loss: 0.4072, train_acc: 0.8181, v

```
al_loss: 0.4554,        val_acc: 0.7500
31:      [0s / 0s],             train_loss: 0.4354,      train_acc: 0.7673,        v
al_loss: 0.4591,        val_acc: 0.7500
32:      [0s / 0s],             train_loss: 0.4148,      train_acc: 0.7800,        v
al_loss: 0.4467,        val_acc: 0.7500
33:      [0s / 0s],             train_loss: 0.4032,      train_acc: 0.8177,        v
al_loss: 0.4493,        val_acc: 0.7250
34:      [0s / 0s],             train_loss: 0.3965,      train_acc: 0.8046,        v
al_loss: 0.4551,        val_acc: 0.7250
35:      [0s / 0s],             train_loss: 0.4193,      train_acc: 0.7792,        v
al_loss: 0.4545,        val_acc: 0.7500
36:      [0s / 0s],             train_loss: 0.4187,      train_acc: 0.7734,        v
al_loss: 0.4506,        val_acc: 0.7500
```

In [21]:

```
log.to_pandas()[['train_loss', 'val_loss']].plot()
plt.xlabel('epoch')
_ = plt.ylabel('loss')
```

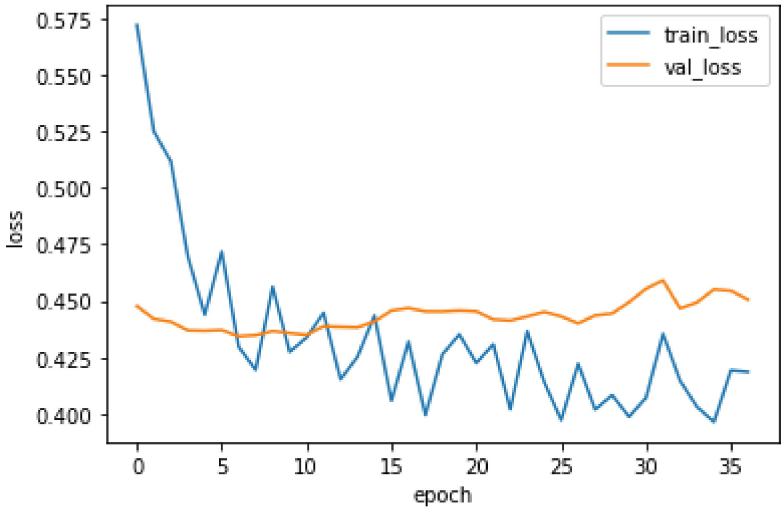

In [79]:

```
preds_train = model.predict(x_train_use, numpy=False).sigmoid().numpy()
preds_test = model.predict(x_test_use, numpy=False).sigmoid().numpy()
print("Train data AUC:")
print(roc_auc_score(y_train_use.flatten(),preds_train.squeeze()))
print("Test data AUC:")
print(roc_auc_score(y_test_use.flatten(),preds_test.squeeze()))
```

```
Train data AUC:
0.7707264957264957
Test data AUC:
0.77
```

In [80]:

```
print("Train data:")
print(model.score_in_batches((x_train_use,y_train_use)))
print("Test data:")
print(model.score_in_batches((x_test_use,y_test_use)))
```

```
Train data:
{'loss': 0.4162150025367737, 'acc': 0.8113207817077637}
Test data:
{'loss': 0.43435677886009216, 'acc': 0.7749999761581421}
```

In [81]:

```
log.to_pandas()[['train_acc', 'val_acc']].plot()
plt.xlabel('epoch')
_ = plt.ylabel('accuracy')
```

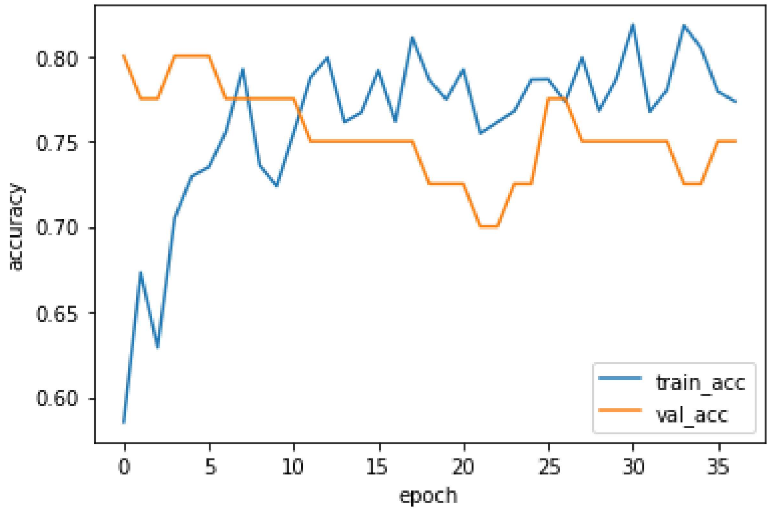

In [82]:

```
auc_train = pd.DataFrame({'Actual': y_train_use.flatten(), 'Predicted': preds_train.squeeze()})
auc_train.head()
```

Out[82]:

	Actual	Predicted
**0**	0.0	0.617593
**1**	1.0	0.599563
**2**	1.0	0.571622
**3**	1.0	0.582445
**4**	1.0	0.617593

In [83]:

```
auc_test = pd.DataFrame({'Actual': y_test_use.flatten(), 'Predicted': preds_test.squeeze()})
auc_test.head()
```

Out[83]:

	Actual	Predicted
**0**	1.0	0.604499
**1**	1.0	0.606305
**2**	1.0	0.573167
**3**	1.0	0.549073
**4**	1.0	0.608318

In [84]:

```
auc_test.to_csv("auc_test.csv",index=False)
auc_train.to_csv("auc_train.csv",index=False)
```

In [85]:

```
model.save_model_weights('myweights.pt')
lz = torch.load('myweights.pt')
print(lz)
```

```
OrderedDict([('net.0.linear.weight', tensor([[-0.0261,  0.1912,  0.3310,  0.3083,
0.1635,  0.3507, -0.6094,  0.0609,
         -0.1091,  0.2160,  0.1913,  0.0198,  0.0313, -0.2745,  0.0382, -0.1096,
          0.2910,  0.3588,  0.3230, -0.3179,  0.0536, -0.0837, -0.2794,  0.0343,
         -0.1820,  0.0659, -0.2268, -0.2512,  0.2144,  0.1516,  0.1528],
        [-0.1556,  0.2122,  0.0792,  0.4084, -0.5649,  0.0096, -0.0774,  0.0228,
         -0.2164, -0.3510,  0.3931, -0.2789, -0.3514, -0.3742,  0.4667,  0.0710,
          0.0042,  0.3877,  0.1088, -0.3377,  0.4110, -0.0862, -0.5418, -0.5793,
         -0.3987, -0.2462, -0.3072,  0.0940,  0.3646, -0.2730,  0.2565],
        [-0.3216,  0.0566,  0.1133, -0.1242,  0.1410,  0.4026,  0.2407, -0.4026,
         -0.4739,  0.2466, -0.0676, -0.0886,  0.0930,  0.1707, -0.4735, -0.4282,
         -0.0685, -0.1479,  0.3785, -0.0643, -0.3479,  0.1811, -0.0396, -0.0087,
         -0.0206,  0.0078,  0.0433,  0.5812, -0.3060,  0.0803, -0.1661],
        [-0.3324, -0.1584,  0.1562,  0.0413, -0.0803, -0.0461, -0.0203, -0.0440,
         -0.2787,  0.1848, -0.3821,  0.1132,  0.0323, -0.2227,  0.2052,  0.1898,
          0.0708, -0.3454,  0.4547, -0.4656,  0.3859,  0.0808,  0.0098, -0.0982,
         -0.0142,  0.2130, -0.4016, -0.1226, -0.0259,  0.3057, -0.0987],
        [ 0.5057,  0.2065, -0.1790,  0.1009, -0.0750,  0.0521, -0.1571,  0.6653,
          0.1938,  0.1510,  0.0713, -0.7428, -0.2344,  0.0644, -0.0346,  0.2629,
         -0.0159, -0.5530,  0.0517,  0.0735, -0.1829,  0.0915,  0.2179,  0.4465,
         -0.0807,  0.0661,  0.3893, -0.2723, -0.0902,  0.4946,  0.0052],
        [ 0.6171, -0.0683, -0.2061,  0.0791,  0.4575,  0.0396, -0.1251,  0.0831,
          0.0210,  0.6841,  0.0571, -0.1829,  0.0446,  0.2977, -0.5439,  0.3126,
         -0.2928, -0.0515, -0.2556, -0.2441,  0.0524, -0.1917,  0.1897,  0.2894,
          0.3401,  0.2440,  0.3524, -0.1732,  0.1085,  0.2168,  0.0529],
        [-0.3556,  0.1665, -0.0182,  0.3874, -0.4090,  0.0081,  0.2442,  0.0165,
         -0.3972, -0.3388,  0.1218, -0.1136, -0.3701,  0.4910,  0.1125, -0.4271,
         -0.1246,  0.0812, -0.2156,  0.2966,  0.1707,  0.2628, -0.2991, -0.2196,
         -0.0043, -0.0968,  0.1884,  0.3276, -0.4212, -0.2618, -0.0252],
        [-0.1171, -0.1484,  0.2027, -0.0599, -0.2956,  0.4812,  0.1686,  0.0429,
          0.4386, -0.2821, -0.1664, -0.1451,  0.0721, -0.0124, -0.0183,  0.6872,
         -0.1431,  0.0220, -0.1034,  0.0467, -0.0124,  0.2396,  0.2236,  0.7747,
         -0.1794, -0.3780, -0.1378, -0.5328, -0.1449, -0.0273,  0.1356],
        [-0.0523,  0.0504,  0.1545,  0.6214,  0.1829, -0.1430,  0.2486, -0.4468,
         -0.6358, -0.4671,  0.5744, -0.3324, -0.1558, -0.2306,  0.1496, -0.1891,
         -0.2290,  0.0009, -0.0675,  0.1726, -0.4093,  0.1225, -0.3350, -0.1627,
          0.3529, -0.5814,  0.1182,  0.2079,  0.1227, -0.0161,  0.4915],
        [-0.0244,  0.2535, -0.1463, -0.2447, -0.0640, -0.1569,  0.0191, -0.1755,
          0.0126, -0.0023,  0.1284, -0.3005, -0.1009,  0.0493,  0.1747, -0.1106,
          0.2233, -0.3601, -0.7139,  0.1803, -0.1880, -0.6561,  0.0340,  0.0239,
         -0.4681, -0.1979,  0.3830,  0.1689, -0.4819, -0.2580, -0.0189],
        [ 0.0123, -0.0672, -0.7857,  0.3998, -0.0272,  0.7440, -0.1669,  0.1976,
         -0.0979,  0.0602,  0.2336, -0.2414, -0.0859, -0.3917,  0.2507, -0.0514,
          0.3001, -0.0735,  0.2034, -0.1164, -0.2968, -0.1818, -0.2427, -0.3576,
         -0.0132, -0.3744, -0.0259, -0.0145,  0.2629,  0.1088, -0.1445],
        [-0.1591,  0.6967,  0.3614, -0.1228,  0.0253,  0.0445,  0.2420,  0.5345,
         -0.0321, -0.4918,  0.4204, -0.1725, -0.0598, -0.0150,  0.0584, -0.5703,
         -0.2477, -0.2146,  0.0181, -0.0509, -0.2438,  0.0866,  0.2871, -0.7476,
         -0.3039,  0.0525,  0.1257,  0.1164,  0.0013,  0.1594, -0.0145],
        [-0.0116, -0.2550,  0.3287, -0.1932,  0.1351,  0.0947,  0.1443, -0.4394,
          0.1381, -0.0779, -0.3749, -0.0983,  0.1699, -0.0402,  0.2907, -0.0167,
         -0.4559, -0.2251, -0.3966,  0.1337, -0.1039,  0.3718,  0.1620,  0.0928,
          0.2451,  0.3811,  0.3029,  0.2336, -0.3198, -0.0484,  0.2005],
        [ 0.0472,  0.2454, -0.1396,  0.0548,  0.2769, -0.3975, -0.3616, -0.1298,
         -0.7511, -0.2150,  0.0260, -0.0525, -0.2434, -0.0754, -0.1438, -0.1246,
         -0.2532, -0.0929, -0.1684,  0.0101,  0.3098, -0.2483, -0.2676, -0.3133,
          0.2297,  0.4510, -0.0448,  0.2734,  0.6275, -0.0786,  0.0134],
        [-0.1168, -0.2305,  0.1111,  0.1493,  0.3278, -0.0583,  0.0128, -0.3286,
         -0.4697,  0.7039,  0.1418, -0.3613, -0.1855,  0.0966, -0.6564,  0.2724,
         -0.2306,  0.1830, -0.0915,  0.3071, -0.0621, -0.2906, -0.0983,  0.0128,
         -0.5793,  0.0278, -0.1764, -0.3537,  0.1967, -0.2877,  0.4496],
```

```
        [ 0.3091, -0.2718, -0.3571, -0.2074, -0.0976,  0.2949,  0.3864,  0.2167,
          0.3029,  0.0275, -0.1999, -0.0200, -0.0686,  0.0602,  0.1919,  0.0818,
          0.1465, -0.3531, -0.1623, -0.0594, -0.0849,  0.4164, -0.0282, -0.1697,
         -0.0166, -0.0484, -0.3099, -0.0782,  0.2413,  0.2272, -0.0336]])), ('ne
t.0.linear.bias', tensor([-0.1782, -0.1824,  0.0843,  0.1026, -0.0531,  0.1303, -
0.1076,  0.0639,
         0.0266, -0.0650, -0.1351, -0.1029, -0.1023,  0.0836, -0.0704, -0.080
3])), ('net.0.batch_norm.weight', tensor([0.9295, 1.0108, 0.8827, 0.9214, 1.0587,
0.8696, 0.9399, 1.1375, 1.0779,
        1.0161, 0.9557, 0.9663, 0.9725, 1.0471, 0.9958, 1.1263])), ('net.0.batch_
norm.bias', tensor([ 0.0848, -0.0227,  0.0448,  0.0325,  0.0708,  0.1036,  0.000
4, -0.0593,
        -0.1015, -0.1099,  0.0021,  0.0854,  0.0770, -0.0987,  0.0598,  0.059
6])), ('net.0.batch_norm.running_mean', tensor([0.1336, 0.0963, 0.8054, 0.5093,
0.4819, 0.8539, 0.4265, 0.2443, 0.3120,
        0.0068, 0.0522, 0.3057, 0.6914, 0.7961, 0.0356, 0.4151])), ('net.0.batch_
norm.running_var', tensor([0.2569, 0.1410, 0.7632, 0.2991, 0.8676, 0.8668, 0.298
2, 0.5468, 0.4992,
        0.0267, 0.0935, 0.7387, 0.4921, 0.5556, 0.0680, 0.4550])), ('net.0.batch_
norm.num_batches_tracked', tensor(35)), ('net.1.linear.weight', tensor([[ 0.3329,
0.1580,  0.0078, -0.5310, -0.0791, -0.2291, -0.4120,  0.3710,
          0.0674,  0.6538,  0.0012,  0.3210, -0.1025, -0.2791,  0.3111,  0.5077],
        [-0.1136, -0.8906,  0.2864,  0.2115, -0.3121,  0.7889, -0.4419, -0.5647,
         -0.3551,  0.1669, -0.0033, -0.0325, -0.0037, -0.6416,  0.4017, -0.1085],
        [-0.6031, -0.8191, -0.0622, -0.0119,  0.0844, -0.5519, -0.2568,  0.1220,
          0.1110,  0.0945, -0.2579, -0.0065, -0.2717, -0.6187,  0.0770,  0.3024],
        [ 0.0515,  0.1155,  0.0449, -0.1839,  0.2763, -0.0829,  0.4230,  0.0484,
          0.0514,  0.0790,  0.2433,  0.2058,  0.3336,  0.1823, -0.0302, -0.116
0]])), ('net.1.linear.bias', tensor([0.0909, 0.2051, 0.0010, 0.1484])), ('net.1.b
atch_norm.weight', tensor([1.0819, 0.9667, 0.9495, 1.0124])), ('net.1.batch_norm.
bias', tensor([ 0.0593, -0.0628, -0.0369, -0.0706])), ('net.1.batch_norm.running_
mean', tensor([0.6801, 1.0883, 0.5972, 0.4186])), ('net.1.batch_norm.running_va
r', tensor([2.3105, 1.6089, 0.7816, 0.5535])), ('net.1.batch_norm.num_batches_tra
cked', tensor(35)), ('net.2.linear.weight', tensor([[ 0.7677, -0.1703,  1.1562, -
0.1619],
        [ 1.0178, -0.9461,  0.1425,  0.1483]])), ('net.2.linear.bias', tensor([0.
4059, 0.1404])), ('net.2.batch_norm.weight', tensor([0.8544, 0.7877])), ('net.2.b
atch_norm.bias', tensor([-0.2634, -0.1641])), ('net.2.batch_norm.running_mean', t
ensor([0.8382, 0.7109])), ('net.2.batch_norm.running_var', tensor([2.3982, 1.837
4])), ('net.2.batch_norm.num_batches_tracked', tensor(35)), ('net.3.weight', tens
or([[-0.2811, -0.4770]]))])
```

In [ ]:

```

```
