# Supplementary material for: Neural network-based model for evaluating inert nodules and volume doubling time in T1 lung adenocarcinoma: a nested case−control study
Source: Front Oncol. 2023 May 24;13:1037052. doi: 10.3389/fonc.2023.1037052 (PMC10244560; doi:10.3389/fonc.2023.1037052)
Supplement: Supplementary file 2 [file DataSheet_2.pdf]

OrderedDict([('net.0.linear.weight', tensor([[-0.0261, 0.1912, 0.3310, 0.3083, 0.1635,
0.3507, -0.6094, 0.0609,
-0.1091, 0.2160, 0.1913, 0.0198, 0.0313, -0.2745, 0.0382, -0.1096,
0.2910, 0.3588, 0.3230, -0.3179, 0.0536, -0.0837, -0.2794, 0.0343,
-0.1820, 0.0659, -0.2268, -0.2512, 0.2144, 0.1516, 0.1528],
[-0.1556, 0.2122, 0.0792, 0.4084, -0.5649, 0.0096, -0.0774, 0.0228,
-0.2164, -0.3510, 0.3931, -0.2789, -0.3514, -0.3742, 0.4667, 0.0710,
0.0042, 0.3877, 0.1088, -0.3377, 0.4110, -0.0862, -0.5418, -0.5793,
-0.3987, -0.2462, -0.3072, 0.0940, 0.3646, -0.2730, 0.2565],
[-0.3216, 0.0566, 0.1133, -0.1242, 0.1410, 0.4026, 0.2407, -0.4026,
-0.4739, 0.2466, -0.0676, -0.0886, 0.0930, 0.1707, -0.4735, -0.4282,
-0.0685, -0.1479, 0.3785, -0.0643, -0.3479, 0.1811, -0.0396, -0.0087,
-0.0206, 0.0078, 0.0433, 0.5812, -0.3060, 0.0803, -0.1661],
[-0.3324, -0.1584, 0.1562, 0.0413, -0.0803, -0.0461, -0.0203, -0.0440,
-0.2787, 0.1848, -0.3821, 0.1132, 0.0323, -0.2227, 0.2052, 0.1898,
0.0708, -0.3454, 0.4547, -0.4656, 0.3859, 0.0808, 0.0098, -0.0982,
-0.0142, 0.2130, -0.4016, -0.1226, -0.0259, 0.3057, -0.0987],
[0.5057, 0.2065, -0.1790, 0.1009, -0.0750, 0.0521, -0.1571, 0.6653,
0.1938, 0.1510, 0.0713, -0.7428, -0.2344, 0.0644, -0.0346, 0.2629,
-0.0159, -0.5530, 0.0517, 0.0735, -0.1829, 0.0915, 0.2179, 0.4465,
-0.0807, 0.0661, 0.3893, -0.2723, -0.0902, 0.4946, 0.0052],
[0.6171, -0.0683, -0.2061, 0.0791, 0.4575, 0.0396, -0.1251, 0.0831,
0.0210, 0.6841, 0.0571, -0.1829, 0.0446, 0.2977, -0.5439, 0.3126,
-0.2928, -0.0515, -0.2556, -0.2441, 0.0524, -0.1917, 0.1897, 0.2894,
0.3401, 0.2440, 0.3524, -0.1732, 0.1085, 0.2168, 0.0529],
[-0.3556, 0.1665, -0.0182, 0.3874, -0.4090, 0.0081, 0.2442, 0.0165,
-0.3972, -0.3388, 0.1218, -0.1136, -0.3701, 0.4910, 0.1125, -0.4271,
-0.1246, 0.0812, -0.2156, 0.2966, 0.1707, 0.2628, -0.2991, -0.2196,
-0.0043, -0.0968, 0.1884, 0.3276, -0.4212, -0.2618, -0.0252],
[-0.1171, -0.1484, 0.2027, -0.0599, -0.2956, 0.4812, 0.1686, 0.0429,
0.4386, -0.2821, -0.1664, -0.1451, 0.0721, -0.0124, -0.0183, 0.6872,
-0.1431, 0.0220, -0.1034, 0.0467, -0.0124, 0.2396, 0.2236, 0.7747,
-0.1794, -0.3780, -0.1378, -0.5328, -0.1449, -0.0273, 0.1356],
[-0.0523, 0.0504, 0.1545, 0.6214, 0.1829, -0.1430, 0.2486, -0.4468,
-0.6358, -0.4671, 0.5744, -0.3324, -0.1558, -0.2306, 0.1496, -0.1891,
-0.2290, 0.0009, -0.0675, 0.1726, -0.4093, 0.1225, -0.3350, -0.1627,
0.3529, -0.5814, 0.1182, 0.2079, 0.1227, -0.0161, 0.4915],
[-0.0244, 0.2535, -0.1463, -0.2447, -0.0640, -0.1569, 0.0191, -0.1755,
0.0126, -0.0023, 0.1284, -0.3005, -0.1009, 0.0493, 0.1747, -0.1106,
0.2233, -0.3601, -0.7139, 0.1803, -0.1880, -0.6561, 0.0340, 0.0239,
-0.4681, -0.1979, 0.3830, 0.1689, -0.4819, -0.2580, -0.0189],
[0.0123, -0.0672, -0.7857, 0.3998, -0.0272, 0.7440, -0.1669, 0.1976,
-0.0979, 0.0602, 0.2336, -0.2414, -0.0859, -0.3917, 0.2507, -0.0514,
0.3001, -0.0735, 0.2034, -0.1164, -0.2968, -0.1818, -0.2427, -0.3576,

-0.0132, -0.3744, -0.0259, -0.0145, 0.2629, 0.1088, -0.1445],
[-0.1591, 0.6967, 0.3614, -0.1228, 0.0253, 0.0445, 0.2420, 0.5345,
-0.0321, -0.4918, 0.4204, -0.1725, -0.0598, -0.0150, 0.0584, -0.5703,
-0.2477, -0.2146, 0.0181, -0.0509, -0.2438, 0.0866, 0.2871, -0.7476,
-0.3039, 0.0525, 0.1257, 0.1164, 0.0013, 0.1594, -0.0145],
[-0.0116, -0.2550, 0.3287, -0.1932, 0.1351, 0.0947, 0.1443, -0.4394,
0.1381, -0.0779, -0.3749, -0.0983, 0.1699, -0.0402, 0.2907, -0.0167,
-0.4559, -0.2251, -0.3966, 0.1337, -0.1039, 0.3718, 0.1620, 0.0928,
0.2451, 0.3811, 0.3029, 0.2336, -0.3198, -0.0484, 0.2005],
[0.0472, 0.2454, -0.1396, 0.0548, 0.2769, -0.3975, -0.3616, -0.1298,
-0.7511, -0.2150, 0.0260, -0.0525, -0.2434, -0.0754, -0.1438, -0.1246,
-0.2532, -0.0929, -0.1684, 0.0101, 0.3098, -0.2483, -0.2676, -0.3133,
0.2297, 0.4510, -0.0448, 0.2734, 0.6275, -0.0786, 0.0134],
[-0.1168, -0.2305, 0.1111, 0.1493, 0.3278, -0.0583, 0.0128, -0.3286,
-0.4697, 0.7039, 0.1418, -0.3613, -0.1855, 0.0966, -0.6564, 0.2724,
-0.2306, 0.1830, -0.0915, 0.3071, -0.0621, -0.2906, -0.0983, 0.0128,
-0.5793, 0.0278, -0.1764, -0.3537, 0.1967, -0.2877, 0.4496],
[0.3091, -0.2718, -0.3571, -0.2074, -0.0976, 0.2949, 0.3864, 0.2167,
0.3029, 0.0275, -0.1999, -0.0200, -0.0686, 0.0602, 0.1919, 0.0818,
0.1465, -0.3531, -0.1623, -0.0594, -0.0849, 0.4164, -0.0282, -0.1697,
-0.0166, -0.0484, -0.3099, -0.0782, 0.2413, 0.2272, -0.0336]])), ('net.0.linea
r.bias', tensor([-0.1782, -0.1824, 0.0843, 0.1026, -0.0531, 0.1303, -0.1076, 0.0639,
0.0266, -0.0650, -0.1351, -0.1029, -0.1023, 0.0836, -0.0704, -0.0803])), ('ne
t.0.batch_norm.weight', tensor([0.9295, 1.0108, 0.8827, 0.9214, 1.0587, 0.8696, 0.9399,
1.1375, 1.0779,
1.0161, 0.9557, 0.9663, 0.9725, 1.0471, 0.9958, 1.1263])), ('net.0.batch_norm.
bias', tensor([0.0848, -0.0227, 0.0448, 0.0325, 0.0708, 0.1036, 0.0004, -0.0593,
-0.1015, -0.1099, 0.0021, 0.0854, 0.0770, -0.0987, 0.0598, 0.0596])), ('n
et.0.batch_norm.running_mean', tensor([0.1336, 0.0963, 0.8054, 0.5093, 0.4819, 0.8539,
0.4265, 0.2443, 0.3120,
0.0068, 0.0522, 0.3057, 0.6914, 0.7961, 0.0356, 0.4151])), ('net.0.batch_norm.r
unning_var', tensor([0.2569, 0.1410, 0.7632, 0.2991, 0.8676, 0.8668, 0.2982, 0.5468, 0.
4992,
0.0267, 0.0935, 0.7387, 0.4921, 0.5556, 0.0680, 0.4550])), ('net.0.batch_norm.
num_batches_tracked', tensor(35)), ('net.1.linear.weight', tensor([[0.3329, 0.1580, 0.00
78, -0.5310, -0.0791, -0.2291, -0.4120, 0.3710,
0.0674, 0.6538, 0.0012, 0.3210, -0.1025, -0.2791, 0.3111, 0.5077],
[-0.1136, -0.8906, 0.2864, 0.2115, -0.3121, 0.7889, -0.4419, -0.5647,
-0.3551, 0.1669, -0.0033, -0.0325, -0.0037, -0.6416, 0.4017, -0.1085],
[-0.6031, -0.8191, -0.0622, -0.0119, 0.0844, -0.5519, -0.2568, 0.1220,
0.1110, 0.0945, -0.2579, -0.0065, -0.2717, -0.6187, 0.0770, 0.3024],
[0.0515, 0.1155, 0.0449, -0.1839, 0.2763, -0.0829, 0.4230, 0.0484,
0.0514, 0.0790, 0.2433, 0.2058, 0.3336, 0.1823, -0.0302, -0.1160]])),
('net.1.linear.bias', tensor([0.0909, 0.2051, 0.0010, 0.1484])), ('net.1.batch_norm.weight', t

ensor([1.0819, 0.9667, 0.9495, 1.0124])), ('net.1.batch_norm.bias', tensor([0.0593, -0.06
28, -0.0369, -0.0706])), ('net.1.batch_norm.running_mean', tensor([0.6801, 1.0883, 0.597
2, 0.4186])), ('net.1.batch_norm.running_var', tensor([2.3105, 1.6089, 0.7816, 0.5535])),
('net.1.batch_norm.num_batches_tracked', tensor(35)), ('net.2.linear.weight', tensor([[0.767
7, -0.1703, 1.1562, -0.1619],
[1.0178, -0.9461, 0.1425, 0.1483]])), ('net.2.linear.bias', tensor([0.4059, 0.14
04])), ('net.2.batch_norm.weight', tensor([0.8544, 0.7877])), ('net.2.batch_norm.bias', tenso
r([-0.2634, -0.1641])), ('net.2.batch_norm.running_mean', tensor([0.8382, 0.7109])), ('net.
2.batch_norm.running_var', tensor([2.3982, 1.8374])), ('net.2.batch_norm.num_batches_trac
ked', tensor(35)), ('net.3.weight', tensor([[-0.2811, -0.4770]]))])
