# Supplementary material for: Neural network-based model for evaluating inert nodules and volume doubling time in T1 lung adenocarcinoma: a nested case−control study
Source: Front Oncol. 2023 May 24;13:1037052. doi: 10.3389/fonc.2023.1037052 (PMC10244560; doi:10.3389/fonc.2023.1037052)
Supplement: Supplementary file 3 [file DataSheet_3.pdf]

In [1]:

```
from sklearn.model_selection import train_test_split
from sklearn.preprocessing import PolynomialFeatures
import pandas  as pd
import numpy as np
import matplotlib.pyplot as plt
```

In [2]:

```
df_raw = pd.read_csv("lasso.csv",na_values="Unknown")
df_raw = df_raw.dropna()
df_raw.head()
```

Out[2]:

	Age	Sex	Smoke	Location	Node classification	Minimum of CT value	Maximum of CT value	Average of CT value(HU)	Kurtosis	Ske
**0**	50	1	1	3	1	-765	22	-563.0	0.19	
**1**	43	1	1	3	3	-361	54	-146.0	-0.05	
**2**	70	1	1	3	1	-528	25	-341.0	0.31	
**3**	37	1	1	3	1	-591	-8	-382.0	0.21	
**4**	71	1	1	3	1	-538	78	-258.0	0.01	

In [3]:

```
# take out y
df_raw_y = df_raw["VDT"]
df_raw_y.head()
```

Out[3]:

```
0     1162.0
1      589.0
2     1226.0
3      929.0
4      656.0
Name: VDT, dtype: float64
```

In [4]:

```
# take out x
df_raw_x = df_raw[['Age', 'Sex', 'Smoke', 'Location', 'Node classification',
       'Minimum of CT value', 'Maximum of CT value', 'Average of CT value(HU)',
       'Kurtosis', 'Skewness', 'CT longest diameter', 'CT shortes diameter',
       'CT average diameter', 'Volume of nodules',
       'Proportion of solid ingredients', 'Shape of nodules', 'Lobulation',
       'Spiculation', 'Pleural retraction']]
df_raw_x.head()
```

Out[4]:

	Age	Sex	Smoke	Location	Node classification	Minimum of CT value	Maximum of CT value	Average of CT value(HU)	Kurtosis	Ske
**0**	50	1	1	3	1	-765	22	-563.0	0.19	
**1**	43	1	1	3	3	-361	54	-146.0	-0.05	
**2**	70	1	1	3	1	-528	25	-341.0	0.31	
**3**	37	1	1	3	1	-591	-8	-382.0	0.21	
**4**	71	1	1	3	1	-538	78	-258.0	0.01	

In [5]:

```
x_train, x_test, y_train, y_test = train_test_split(df_raw_x, df_raw_y, test_size=0.2, random_sta
```

In [6]:

```
from  sklearn.neural_network import MLPRegressor
```

In [7]:

```
nn = MLPRegressor(max_iter=10000,
                  hidden_layer_sizes=(32,8),
                  learning_rate_init=.01,
                 )
nn.fit(x_train,y_train)
```

Out[7]:

```
MLPRegressor(hidden_layer_sizes=(32, 8), learning_rate_init=0.01,
             max_iter=10000)
```

In [8]:

```
nn.score(x_train, y_train)
```

Out[8]:

0.8007584066398086

In [9]:

```
nn.score(x_test, y_test)
```

Out[9]:

```
0.6268093376200479
```

In [10]:

```
plt.plot(nn.loss_curve_)
```

Out[10]:

```
[<matplotlib.lines.Line2D at 0x23093829730>]
```

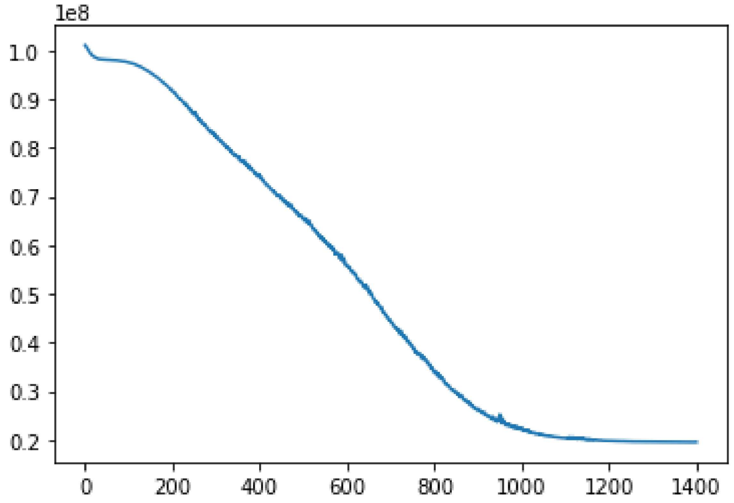

In [11]:

```
nn.intercepts_
```

Out[11]:

```
[array([ 3.45031158,  3.88599435, -0.13713584,  3.83534451,  3.09815482,
         4.15136592,  5.22283368,  3.62084355, -0.82497452, -0.18956587,
         0.00936407,  5.41274568, -0.47161109, -1.26028372, -0.31072794,
         0.65598936, -0.42727891,  3.401332  ,  4.01477504,  4.91968953,
        -0.35793693,  3.70912048,  4.038652  ,  4.16532846,  3.43444221,
        -3.438274  , -0.43160343, -1.86141996, -1.10629426,  3.69651112,
         3.01355019,  3.31306216]),
 array([ 0.25294212,  1.9662303 , -0.28462409,  0.11236097, -0.3653688 ,
         1.56432267, -0.26521274,  0.17734035]),
 array([11.60128294])]
```

In [12]:

```
nn.coefs_
```

Out[12]:

```
[array([[ 5.73271456e+00, -1.50264905e+00, -7.49952692e-02,
         -1.23384151e+00, -1.76530290e+00, -1.54553849e+00,
         -2.09992623e+00, -1.16468368e+00, -8.49283648e-01,
         -1.28748472e-01, -3.89259674e-01, -3.13018527e+00,
         -1.21629999e+00,  2.72204760e+00, -2.98793833e-01,
         -1.52393586e-01, -2.50120558e-01, -1.33866218e+00,
         -1.59414688e+00, -2.40065831e+00, -8.14157914e-01,
         -1.67395627e+00, -1.69734982e+00, -1.94891425e+00,
         -1.49005769e+00,  2.60900484e+00, -2.61782418e-01,
         -7.62927311e-01, -8.02434704e-01, -2.45762051e+00,
         -1.41922260e+00, -2.08563625e+00],
        [ 8.54049075e+00, -5.91858178e+00, -1.26028297e-01,
         -6.36629670e+00, -8.29863479e-01, -5.79890917e+00,
         -3.71334837e+00, -2.83700195e+00,  4.06250427e+00,
         -1.26099470e-01, -4.20709371e-01, -4.44426522e+00,
          4.08490098e+00, -2.96183230e+00,  2.43042760e-01,
         -2.34446567e+00, -3.30562011e+00, -5.92192890e+00,
         -2.13619293e+00, -4.05556976e+00,  4.29520440e+00,
```

In [13]:

```
import pickle
pickle.dump(nn, open("nn.sav", 'wb'))
```

In [14]:

```
train = pd.concat([x_train, y_train], axis=1)
test = pd.concat([x_test, y_test], axis=1)
```

In [15]:

```
train.head()
```

Out[15]:

	Age	Sex	Smoke	Location	Node classification	Minimum of CT value	Maximum of CT value	Average of CT value(HU)	Kurtosis	S
**100**	44	2	2	1	1	-702	-153	-559.0	0.39	
**166**	42	1	1	3	2	-817	407	-632.0	1.12	
**148**	69	1	2	3	2	-770	-186	-650.0	0.54	
**114**	39	1	1	3	2	-672	-19	-468.0	0.91	
**86**	66	1	1	1	2	-781	-105	-685.0	1.57	

In [16]:

```
train.iloc[:,-1]
```

Out[16]:

```
100     430.0
166     640.0
148    2767.0
114     509.0
86     1082.0
        ...
185    2738.0
179    2149.0
113    1275.0
175     837.0
126    -813.0
Name: VDT, Length: 159, dtype: float64
```

In [ ]:

```

```
