# Supplementary material for: Neural network-based model for evaluating inert nodules and volume doubling time in T1 lung adenocarcinoma: a nested case−control study
Source: Front Oncol. 2023 May 24;13:1037052. doi: 10.3389/fonc.2023.1037052 (PMC10244560; doi:10.3389/fonc.2023.1037052)
Supplement: Supplementary file 4 [file DataSheet_4.pdf]

```
nn.intercepts_
[array([ 3.45031158,    3.88599435, -0.13713584,    3.83534451,    3.09815482,
         4.15136592,    5.22283368,    3.62084355, -0.82497452, -0.18956587,
         0.00936407,    5.41274568, -0.47161109, -1.26028372, -0.31072794,
         0.65598936, -0.42727891,    3.401332    ,    4.01477504,    4.91968953,
        -0.35793693,    3.70912048,    4.038652    ,    4.16532846,    3.43444221,
        -3.438274    , -0.43160343, -1.86141996, -1.10629426,    3.69651112,
         3.01355019,    3.31306216]),
 array([ 0.25294212,    1.9662303 , -0.28462409,    0.11236097, -0.3653688 ,
         1.56432267, -0.26521274,    0.17734035]),
 array([11.60128294])
```

```
nn.coefs_
[array([[ 5.73271456e+00, -1.50264905e+00, -7.49952692e-02,
         -1.23384151e+00, -1.76530290e+00, -1.54553849e+00,
         -2.09992623e+00, -1.16468368e+00, -8.49283648e-01,
         -1.28748472e-01, -3.89259674e-01, -3.13018527e+00,
         -1.21629999e+00,    2.72204760e+00, -2.98793833e-01,
         -1.52393586e-01, -2.50120558e-01, -1.33866218e+00,
         -1.59414688e+00, -2.40065831e+00, -8.14157914e-01,
         -1.67395627e+00, -1.69734982e+00, -1.94891425e+00,
         -1.49005769e+00,    2.60900484e+00, -2.61782418e-01,
         -7.62927311e-01, -8.02434704e-01, -2.45762051e+00,
         -1.41922260e+00, -2.08563625e+00],
        [ 8.54049075e+00, -5.91858178e+00, -1.26028297e-01,
         -6.36629670e+00, -8.29863479e-01, -5.79890917e+00,
         -3.71334837e+00, -2.83700195e+00,    4.06250427e+00,
         -1.26099470e-01, -4.20709371e-01, -4.44426522e+00,
          4.08490098e+00, -2.96183230e+00,    2.43042760e-01,
         -2.34446567e+00, -3.30562011e+00, -5.92192890e+00,
         -2.13619293e+00, -4.05556976e+00,    4.29520440e+00,
         -4.88800510e+00, -4.21967222e+00, -4.86473402e+00,
         -3.74451902e+00,    2.30574838e+00,    6.42748708e-02,
          4.28666186e+00,    3.56693928e+00,    2.02100954e-01,
         -4.84721660e+00, -2.10958712e+00],
        [-1.94898451e-02,    1.48596976e+00,    3.00940987e-02,
          1.88811396e+00,    1.65525615e+00,    1.41786211e+00,
          1.93992732e+00,    2.58959153e+00, -7.02646402e-01,
         -1.32413961e-01,    2.19109934e-01,    1.94170519e+00,
         -2.53455767e-01, -3.59341258e-01,    8.97295123e-02,
          9.07348911e-01, -9.41785644e-01,    1.30987128e+00,
          1.00000781e+00,    1.49815455e+00, -2.36334203e-01,
          8.93592053e-01,    1.71056894e+00,    1.52021667e+00,
```

7.65397916e-01, -2.40502334e-01, 1.59196771e-01,
-1.55443018e+00, -4.66183257e-01, 1.33218171e+00,
9.71109134e-01, 1.80484942e+00],
[9.77280102e+00, -3.90222695e+00, -2.22300041e-01,
-3.98579248e+00, -2.58230453e+00, -3.61297512e+00,
-2.23639975e+00, 4.29420058e+00, 3.71722318e+00,
-1.63537080e-01, 1.94741526e-01, -1.43048828e+00,
4.62471493e+00, -3.61841396e+00, 4.41145391e-02,
-1.02928721e+00, 7.13439066e-01, -3.12232046e+00,
-2.86635555e+00, -1.88015146e+00, 4.53922175e+00,
-4.34052977e+00, -3.81830904e+00, -3.80842130e+00,
-3.41625143e+00, 2.74665131e+00, 1.98730717e-01,
-1.76707419e-01, 3.55607289e+00, -2.12147544e+00,
-3.23028116e+00, -2.33148489e+00],
[-4.72712920e+00, 1.41632965e+01, -3.17411793e-01,
1.41110049e+01, 1.20322237e+01, 1.41234454e+01,
1.27269421e+01, 1.38621904e+01, -4.62783427e+00,
-1.33166919e-01, -3.37258892e-01, 1.30120715e+01,
-4.71408174e+00, -5.37811508e+00, 2.73991452e-01,
2.22512920e-03, -4.80461976e-01, 1.32256751e+01,
1.27256459e+01, 1.32277695e+01, -4.67428738e+00,
1.38713242e+01, 1.39142058e+01, 1.42707190e+01,
1.27865965e+01, -1.15138916e+01, -3.66174636e-01,
-6.08492073e+00, -4.82938906e+00, 1.25055574e+01,
1.26860542e+01, 1.25892461e+01],
[-1.55744645e+00, 3.58498814e-03, 1.64623742e-01,
4.01559916e-02, -3.90983890e-01, 3.11393463e-01,
1.45797377e-01, -2.46059174e-01, 1.69866435e-01,
1.52772719e-01, 1.63121906e-01, 6.26423005e-01,
7.56811812e-02, 1.90949513e-01, 2.57246698e-01,
4.90674799e-01, -7.70016111e-01, 1.16043896e-01,
-1.85408960e-01, 3.21773220e-01, -2.87878641e-02,
4.29598050e-02, 1.10490652e-01, 2.01761391e-01,
8.83487870e-02, -9.28282219e-01, 4.21512232e-01,
1.11184489e+00, 3.09264299e-01, -2.60854095e-01,
1.89010578e-02, -1.45637502e-01],
[5.21065845e+00, 8.10684808e-01, -1.10772194e-01,
7.83098329e-01, 2.27014827e-01, 1.38030876e+00,
1.41642450e+00, -1.83463887e+00, 2.56022742e+00,
-4.39851598e-02, -1.78227119e-01, 2.56692882e+00,
2.43941618e+00, -2.97973674e+00, 1.08317870e-01,
1.98489505e+00, 1.29154620e-01, 1.07135439e+00,
6.45081057e-01, 1.40584903e+00, 2.31801807e+00,
-1.19325053e+00, -1.08756902e+00, -1.36993375e+00,

```
 -2.30449193e+00, -1.27022821e+00,  7.36654464e-02,
  7.65930551e-01,  2.40820090e+00, -1.31591924e-01,
  1.92000505e-01,  5.69822560e-01],
[ 2.36404712e+00,  2.30772441e-01,  1.01122604e-01,
  3.02964113e-01, -1.50132286e-01, -4.85675469e-01,
 -4.77823056e-01,  1.02787154e+00, -1.31377130e-01,
  1.40529473e-01,  1.05767115e-01, -6.92800299e-01,
 -6.48213466e-02, -1.71334496e-01,  3.63692819e-01,
  5.88523849e-01,  6.63338546e-01, -2.74985976e-01,
 -4.47042957e-01, -2.83314357e-01,  5.90139274e-02,
 -2.43171601e-01, -3.04734779e-01, -4.04431714e-01,
 -1.82077011e-01, -7.08535798e-01,  3.94460956e-01,
 -1.04662662e-01, -2.46253140e-01, -1.98722156e-01,
  8.56120220e-02, -3.64106485e-01],
[ 9.05255387e+00, -9.91987853e+00,  8.72580205e-03,
 -9.97649642e+00, -7.67326682e+00, -1.03313796e+01,
 -9.54169935e+00, -4.02366448e+00,  5.41729765e+00,
 -1.36781418e-01,  3.98845103e-02, -9.64777502e+00,
  5.86951111e+00,  6.61380613e+00,  1.49540677e-02,
 -6.33198790e-01,  5.72412148e-01, -8.91478964e+00,
 -7.87443951e+00, -9.86499756e+00,  6.02560959e+00,
 -9.33591331e+00, -9.48438358e+00, -9.22192391e+00,
 -8.44701468e+00,  8.08309920e+00,  7.46830172e-02,
  1.96611299e+00,  5.35761723e+00, -7.44363598e+00,
 -8.85420791e+00, -8.56842693e+00],
[ 3.94524229e+00, -1.58417774e+01,  3.08435783e-01,
 -1.62311286e+01, -1.25616408e+01, -1.55668125e+01,
 -1.33376665e+01, -1.69696398e+01,  9.80383052e+00,
 -3.27347838e-02,  3.70624683e-01, -1.37010876e+01,
  9.56169076e+00,  8.85685367e+00, -1.19849945e-01,
 -1.91621506e+00, -1.02915356e+00, -1.41659995e+01,
 -1.35598717e+01, -1.39124802e+01,  9.41392423e+00,
 -1.40517715e+01, -1.44423885e+01, -1.36077574e+01,
 -1.33830816e+01,  1.16537021e+01, -3.52377011e-01,
  5.52530616e+00,  9.52826068e+00, -1.24060545e+01,
 -1.41529154e+01, -1.32672734e+01],
[-5.58713271e+00,  2.03050979e+00, -3.54903448e-01,
  1.80045132e+00,  1.26340597e+00,  1.96698004e+00,
  7.95127819e-01,  2.73712845e+00,  5.99036411e-01,
 -1.38456581e-01, -6.79971819e-02,  1.56580319e+00,
  1.76867137e-01, -1.56762412e+00, -2.51727983e-01,
  2.41202482e+00,  2.13660464e+00,  1.23252406e+00,
  8.34749040e-01,  1.40547937e+00,  5.18445269e-01,
  1.89400617e+00,  1.96113399e+00,  1.35303529e+00,
```

2.00171800e+00, -2.75067738e-01, -9.62978560e-02,
3.85750766e-01, 6.59525964e-01, 1.58642370e+00,
1.38778220e+00, 9.99643966e-01],
[-2.83423561e+00, 1.57915582e+00, -4.37701928e-01,
1.81946797e+00, 7.98719900e-02, 8.26993171e-01,
-7.71255793e-01, 2.55026914e+00, 1.32783878e-01,
-1.54121473e-01, 3.17670821e-02, -1.39619694e+00,
3.32015061e-01, -7.67855707e-01, 2.06628614e-01,
2.56393284e+00, 1.63000327e+00, 6.01018574e-01,
-1.36911794e-01, -2.81966513e-01, 3.50746176e-01,
2.13016053e-01, 3.57258866e-01, -9.75458873e-02,
8.81899329e-02, 1.99083952e+00, 1.28338358e-01,
-1.41080832e-02, 4.38067388e-02, -7.53002538e-01,
1.00129959e+00, 3.77691974e-01],
[-5.61436648e+00, 1.65468933e+00, -3.97114834e-01,
1.81160335e+00, 2.53109601e-01, 6.22612643e-01,
-1.20090125e-01, 2.24767620e+00, 4.27499364e-01,
-1.28268514e-01, -3.45923818e-01, -6.98950366e-01,
-5.90287883e-02, -7.94990307e-01, -2.46458259e-01,
3.21136459e+00, 2.12397477e+00, 7.33558334e-01,
-2.81527028e-01, 7.73740953e-02, 5.40339695e-01,
4.92704113e-01, 9.87094913e-01, 3.10770519e-01,
6.33743191e-01, 1.55381412e+00, 2.55791929e-02,
3.39531991e-01, 1.99222798e-01, -4.93405464e-01,
1.13913196e+00, 3.43199171e-01],
[-1.38737943e+00, 1.30484830e+00, -1.93179810e-01,
1.52039886e+00, -4.79565872e-01, 1.22226381e+00,
1.08633338e+00, 6.01029191e-01, 1.89586736e+00,
-9.11172257e-02, -1.96057084e-02, 3.07489971e+00,
1.79185805e+00, -2.01118968e+00, -3.51142702e-01,
-4.87330857e-01, -2.22315958e+00, 8.93249169e-01,
-2.62403197e-01, 1.87137158e+00, 1.59712527e+00,
-7.69364457e-01, -7.01143876e-01, -7.37165022e-01,
-1.23542608e+00, 1.08382300e+00, -3.30476093e-01,
2.85552692e+00, 1.91268508e+00, -5.11621871e-01,
5.59836772e-01, 6.49076764e-02],
[5.52721048e+00, -9.41351440e-01, 5.16513093e-03,
-7.65894311e-01, -4.57081819e+00, -1.56835777e-01,
-2.18293010e+00, -1.08455504e+01, 1.61641862e+00,
-1.69874373e-01, 2.23111306e-01, -7.15498358e-01,
2.18064115e+00, 6.88581279e+00, 2.16720389e-01,
-4.39998058e+00, -7.40764522e+00, -2.40446106e-01,
-1.50941883e+00, -1.64518760e+00, 1.21709809e+00,
-1.38687167e+01, -1.27932137e+01, -1.44058757e+01,

-1.15651379e+01, 2.47658814e+00, -3.79110422e-01,
-5.17708456e+00, -6.33112688e+00, -7.70828184e+00,
-2.03155409e+00, -1.41319704e+00],
[5.19089318e+00, 4.06018525e+00, -5.46505244e-02,
3.78232250e+00, 3.77141549e+00, 4.86755214e+00,
5.21426075e+00, 3.79227702e+00, -8.63897221e-01,
-1.31899347e-01, -1.66092702e-01, 5.45295996e+00,
-1.10469965e+00, -1.94559419e+00, -1.01961088e-02,
-6.35213595e-02, -5.45994303e-01, 4.46518223e+00,
4.39562417e+00, 5.09973464e+00, -8.65425328e-01,
4.16484763e+00, 4.16076374e+00, 4.71025373e+00,
3.35546316e+00, -4.16357619e+00, 1.67933333e-01,
-1.66878363e+00, -1.05456173e+00, 4.13734085e+00,
3.40552468e+00, 3.64044739e+00],
[-6.22798356e-01, -6.75830501e+00, 1.01905756e-01,
-7.17146397e+00, -4.43969555e+00, -6.84154465e+00,
-6.75848564e+00, -5.44611794e+00, 3.06953145e+00,
-1.41457269e-01, 1.64794107e-01, -6.95785913e+00,
3.22744002e+00, -1.12843249e-01, -3.40648518e-01,
-1.55802238e+00, 1.07257367e+00, -6.84044307e+00,
-5.93062226e+00, -6.53320711e+00, 3.62059158e+00,
-7.04432623e+00, -6.51602510e+00, -7.55572121e+00,
-4.95984123e+00, 6.25549660e+00, -7.26230724e-02,
3.16508288e+00, 3.47256658e+00, -4.08140265e+00,
-5.98779895e+00, -5.38312268e+00],
[-1.95931542e+00, -1.54970636e+00, 1.62225128e-01,
-1.19536742e+00, -1.24240972e+00, -2.32459747e+00,
-2.21235381e+00, -4.34574712e-01, 6.96878830e-01,
-1.25099976e-01, 1.63946486e-01, -3.12354276e+00,
9.32239099e-01, 2.48568367e-01, -1.81635249e-01,
2.46427641e+00, 1.75495427e+00, -1.52940584e+00,
-2.06451118e+00, -2.54115732e+00, 9.22981357e-01,
-1.85837377e+00, -1.86904853e+00, -2.44170513e+00,
-1.07523074e+00, 3.33592192e+00, -4.28226528e-01,
-3.04465671e-01, 7.77468869e-01, -1.70948348e+00,
-1.59818441e+00, -1.33451330e+00],
[1.42171288e+00, 3.33416322e+00, 2.05690682e-01,
3.48840454e+00, 2.76324917e+00, 3.69548328e+00,
4.40722070e+00, 3.37932466e+00, -6.19216106e-01,
-1.29336476e-01, 2.04791278e-01, 4.70066886e+00,
-7.27189549e-01, -1.68780996e+00, -2.82964572e-01,
8.21123626e-01, 1.47857114e+00, 3.19015342e+00,
3.20382199e+00, 3.80525065e+00, -3.42940191e-01,
3.30928076e+00, 3.53335281e+00, 3.84498821e+00,

```
         2.99498074e+00, -2.30979805e+00,  4.43640261e-02,
        -1.78916602e+00, -6.37372007e-01,  2.91859114e+00,
         2.67754715e+00,  3.25575948e+00]]),
 array([[ 1.99538427e-02,  6.23287097e+00, -3.96303853e-02,
         8.10556980e-02,  5.40231044e-34,  5.80754176e+00,
        -6.09431783e-34, -2.41083541e-01],
       [ 6.58637354e-02,  3.30232166e+00,  8.16916840e-02,
         1.97593912e-01, -3.89885923e-01,  4.07611973e+00,
        -4.18171617e-01, -2.47899674e-01],
       [ 2.84679586e-01,  4.15320385e-03, -1.88729528e-34,
         1.08808215e-33, -4.35706571e-33, -1.22195410e-01,
         7.47794544e-03,  2.27368131e-33],
       [-4.71174651e-01,  3.48480928e+00, -4.12063119e-01,
        -2.21818125e-01,  1.91266947e-01,  4.66606735e+00,
        -1.69506501e-01, -1.21095550e-01],
       [-3.75668759e-01,  1.73733191e+00, -1.22739533e-01,
         1.23490695e-01, -1.42299411e-01,  2.76865005e+00,
        -4.32725468e-01, -3.09410188e-35],
       [ 1.96191793e-01,  5.05009925e+00, -4.68664733e-01,
        -2.89076852e-01, -4.23543767e-01,  4.54004403e+00,
        -1.36363976e-01,  1.69031165e-01],
       [-4.26511985e-01,  5.06647760e+00, -5.04087115e-02,
        -5.31223504e-02,  4.16326819e-33,  5.48257747e+00,
        -1.69505645e-01, -3.28574816e-02],
       [-2.27422967e-01,  6.15847837e+00, -2.50677530e-01,
         2.92340959e-01, -3.55495808e-01,  4.07320509e+00,
        -1.69504063e-01, -2.47576556e-01],
       [ 2.56469252e-01, -7.57898862e+00,  3.52714551e-02,
        -8.25940247e-02, -3.45638684e-01, -1.18194381e+01,
         2.23314484e-02,  1.02956587e-01],
       [ 8.90004841e-33, -8.33705375e-02,  8.49430770e-34,
        -1.46916797e-33,  3.00741584e-33,  6.60442077e-02,
         3.00553914e-33, -6.00923344e-34],
       [-3.99567505e-02,  3.24291890e-01,  3.74449812e-33,
         3.00773039e-01,  2.83742400e-01, -2.37773552e-01,
         1.63611921e-33, -3.70618612e-33],
       [ 9.99331983e-33,  6.11008015e+00, -1.22866386e-01,
         3.04715344e-33, -6.18427406e-33,  7.68751559e+00,
        -1.69506537e-01,  1.18021893e-33],
       [ 8.89396442e-02, -7.42604549e+00, -1.22755247e-01,
        -4.50641197e-01,  1.92003945e-01, -1.13580376e+01,
        -2.01615604e-01, -8.22873579e-02],
       [ 1.23518616e-01, -1.18274656e+01,  7.04201636e-33,
         1.86333901e-01,  1.74293258e-01, -7.46400098e+00,
```

1.38587339e-33, -9.42900391e-34],
[3.47169406e-02, -9.89936224e-02, -1.40224426e-01,
-3.76965520e-01, 8.68997484e-33, -2.96056608e-01,
-1.44826390e-34, -8.05937479e-35],
[-3.66001531e-01, -6.97608320e+00, 2.16283706e-01,
-3.58646265e-01, -2.79465101e-01, -1.34090182e+00,
7.15624421e-02, -1.93623667e-01],
[7.08710274e-33, -7.75483407e+00, -1.22728456e-01,
-1.65921842e-33, -3.90982538e-33, -6.48334108e+00,
-2.14307440e-34, 3.92120862e-34],
[-4.14910363e-01, 2.06384149e+00, -2.53616257e-01,
-4.41306175e-01, -7.32204090e-02, 1.44541718e+00,
3.57298538e-02, -1.31187083e-01],
[-1.56624013e-01, 2.69493485e+00, -1.00920779e-01,
-3.64636277e-01, -1.42310228e-01, 2.61236707e+00,
-1.73784266e-01, 5.21399737e-02],
[-2.99222102e-01, 6.97424101e+00, -2.63890232e-01,
-7.94539063e-02, 3.97526794e-34, 9.06358140e+00,
-1.69506550e-01, 5.00541660e-03],
[4.91724068e-33, -7.83001874e+00, -1.22866554e-01,
-6.53163245e-33, -4.13103005e-33, -1.28310041e+01,
-1.69506528e-01, -9.79345777e-34],
[-2.99902154e-01, 6.40355625e+00, 2.95306736e-33,
-1.48084532e-01, -1.42313273e-01, 3.68009983e+00,
1.61376752e-01, 6.31393436e-33],
[1.16436216e-01, 5.75056912e+00, -4.25768676e-01,
-1.58226327e-01, -1.56938139e-01, 3.82711669e+00,
1.79678938e-01, -1.34506345e-33],
[-3.79259293e-01, 6.76417654e+00, -1.22786683e-01,
-1.48087657e-01, -1.42315171e-01, 4.01457223e+00,
-1.33970610e-01, -8.79103875e-35],
[-1.80550995e-02, 3.12865600e+00, 2.28397328e-34,
1.27770774e-01, -1.62607200e-02, 3.11261992e+00,
-2.90693463e-01, -6.80674208e-34],
[-3.41622189e-01, -2.55383078e+00, -5.72164064e-01,
-3.58457735e-01, -1.89661610e-01, -2.31546251e+00,
-2.66278843e-01, -1.19515287e-01],
[1.96428550e-01, -4.39757699e-03, -9.21534349e-02,
-3.65640548e-01, -7.61326225e-33, 1.71887004e-01,
-9.63548960e-33, -1.33532245e-01],
[-4.38521413e-01, -1.35669916e+01, -1.22670722e-01,
-8.36148923e-02, 6.92208729e-02, -1.32809936e+01,
-3.13975068e-01, -1.02192604e-01],
[4.15011560e-05, -8.77668031e+00, -1.23029352e-01,

```
         -2.39799439e-01,  4.69849953e-02, -1.22308349e+01,
         -1.69506498e-01,  5.98627441e-02],
        [-5.16024838e-02,  1.55669638e+00, -1.22866205e-01,
         -3.62338999e-01, -3.35076550e-01,  2.97113331e+00,
          2.11272874e-01,  4.02437021e-34],
        [-2.99124445e-02,  1.81923355e+00, -2.62696222e-01,
          4.38373004e-02, -5.50613426e-01,  2.50975537e+00,
         -1.74812571e-01, -3.58336077e-01],
        [ 2.94024886e-01,  2.40381184e+00, -2.86088230e-01,
          3.64769020e-03,  1.27605524e-01,  2.65968661e+00,
         -1.97456752e-01, -9.43908292e-02]]),
array([[-0.65452453],
        [12.84305127],
        [-0.67653812],
        [-0.2985982 ],
        [-0.45390028],
        [13.36124284],
        [-0.27126804],
        [ 0.21912783]])]
```
